# A systematic review of economic evaluations of pharmacological treatments for active tuberculosis

**DOI:** 10.3389/fpubh.2024.1201512

**Published:** 2024-04-16

**Authors:** Sarosh Nagar, David Nicholls, Dalia Dawoud

**Affiliations:** ^1^National Institute for Health and Care Excellence (NICE), London, United Kingdom; ^2^Department of Chemistry & Department of Economics, Harvard College, Harvard University, Boston, MA, United States; ^3^Faculty of Pharmacy, Cairo University, Cairo, Egypt

**Keywords:** tuberculosis, anti-microbial resistance, public health, economic evaluations, systematic review, cost effectiveness, value attributes

## Abstract

**Objectives:**

The continuing spread of tuberculosis (TB) worldwide, especially drug-resistant TB, poses a major challenge to healthcare systems globally. Addressing this requires appraising the cost effectiveness of existing pharmacological interventions against TB to identify key drivers of cost effectiveness and value and guide pharmaceutical innovation and novel drug regimen development.

**Methods:**

Studies were identified from a search of six database: MEDLINE MEDLINE-In Process, MEDLINE Epub Ahead of Print, EMBASE, Cochrane Database of Systematic Reviews, and Econlit in July 2022. Two reviewers independently assessed all identified studies and reports using pre-defined inclusion/exclusion criteria. Study methodological quality was assessed, data were extracted in standard tables, and results were narratively synthesized.

**Results:**

Overall, 991 studies and 53 HTA reports were identified with 20 studies and 3 HTA reports meeting the inclusion criteria. Quality assessment of the 20 studies identified 4 with minor limitations, while the remainder were assessed as having potentially or very serious limitations. Sixteen studies conducted cost-utility analyses, 6 conducted cost-effectiveness analyses, and 2 conducted cost-comparison analyses with some studies performing multiple analyses. The majority (n = 16) were model-based. Eleven studies analyzed the cost-effectiveness of bedaquiline, 6 compared shorter to longer/standard duration regimens, 2 assessed ethambutol, and 1 assessed delamanid. Key drivers of cost effectiveness were drug costs, the number of TB cases, the portion of cases with sputum culture conversion, treatment delivery costs, and treatment efficacy. Common value elements considered included adverse events, drug resistance, and improving treatment adherence.

**Conclusion:**

Our results suggest that out of the pharmacological treatments assessed, bedaquiline is likely a cost-effective addition to existing treatment regimens/background treatment regimens, while ethambutol is not likely to be. Newer shorter regimens, even if more costly, seem to be more cost-effective compared to longer regimens. These results illustrate the limited number of novel cost-effective pharmacological interventions and highlight a need to develop new drugs/regimens against TB to overcome resistance, taking into account the key drivers of cost effectiveness and other value attributes identified from this review.

## Introduction

Tuberculosis (TB) is a highly infectious bacterial disease caused by the pathogen *Mycobacterium tuberculosis* (1). The disease infects over 10 million individuals worldwide and occurs when the bacterium infects individual’s lungs; however, initially, such infections are latent and asymptomatic as the infection is limited to the granuloma (2, 3). Over time, however, latent TB turns into active TB, resulting in coughing, fever, and other symptoms (4). Diagnosis of the disease usually occurs through a sputum culture, supplemented with additional tuberculin skin tests (TST), interferon-gamma assays (IGAs), and other approaches (5).

The discovery of streptomycin in 1944 opened the door to pharmacological treatments against TB, though initial trials identified that use of streptomycin alone induced antimicrobial resistance (AMR) (6). However, the use of para-aminosalicylic acid (PAS) with streptomycin soon introduced the concept of combination therapy using one or more pharmacological interventions, which was far more effective against the disease (7). Using these combination regimens, common anti-TB drugs, such as isoniazid, rifampin, and ethambutol, successfully reduced global TB burden for several decades (7). The global HIV-AIDS epidemic, however, introduced the risk of HIV-AIDS and TB co-infection, causing a rapid increase in TB prevalence, especially in developing nations (8). New infections were further driven by increasingly prevalent multi-drug resistant tuberculosis (MDR-TB) and extensively drug-resistant tuberculosis (XDR-TB), which rendered traditional pharmacological interventions ineffective (9). A lack of investment in developing new antimicrobials against TB further compounded this problem — with only few products, introduced in the last 45 years against drug-resistant TB (10). The COVID-19 pandemic further disrupted global TB eradication efforts and fueled a surge of infections (11).

In response to this increasing global prevalence of tuberculosis, pharmaceutical companies and governments are trying to accelerate the development of novel treatments against tuberculosis through public-private partnership projects like the European Regimen Accelerator for Tuberculosis (ERA4TB) (12). However, access to these novel regimens will only be possible if they are cost-effective and of demonstrated value compared to currently available regimens. To assess this, Health technology assessment (HTA) agencies, that currently exist in many countries to facilitate evidence-informed decision making regarding the allocation of the scarce health system resources, need to understand the key drivers of cost effectiveness of TB treatments and any additional value attributes.

While there have been several past systematic reviews of economic evaluations of treatments for latent TB or both active and latent TB, reviews that focused specifically on active tuberculosis are much rarer (13, 14). The most recent review by Byun et al. focused on active TB but included both pharmacological and non-pharmacological treatments (15). Additionally, to our knowledge, no reviews of published HTA reports of active TB treatments have been published so far.

Therefore, to inform novel regimen development activities and facilitate future appraisals of such novel pharmacological interventions for active TB, we conducted this systematic review. It aimed to describe economic evaluation approaches used in past studies and HTA reports, identify estimates and key drivers of the cost effectiveness and value attributes of pharmacological interventions against TB.

## Methods

### PICOS

This systematic review qualitatively synthesized past economic evaluations of pharmacological treatments of active TB. A review protocol was developed to define the Population, Intervention, Comparator, Outcomes, Study design (PICOS) of the studies to include. Given the limited time available for this review, which was conducted as part of a summer internship, and to minimize research waste, it was conducted as an update of the most recently published systematic review of economic evaluation on the topic and supplemented by a de-novo review of HTA reports and the gray literature. Thus, we started by conducting a rapid review to identify the most relevant and recent systematic review that aligns with our review protocol to update it. Through this initial stage, we identified that the most recent systematic review of interventions for active TB was the one performed by Byun et al., which identified relevant economic evaluations published until January 1st, 2020 (15).

As the scope of Byun et al. review covered both pharmacological and non-pharmacological treatment strategies and interventions for active TB, we only focused on its included studies involving pharmacological interventions (15). We then conducted a systematic search to identify all relevant economic evaluations published after its search cut-off date until June 2022.

### Search strategy & information sources

The search strategy was designed to identify all economic evaluations of pharmacological treatments of any form of active TB, including multidrug-resistant tuberculosis (MDR-TB) and extensively drug-resistant tuberculosis (XDR-TB). We searched six databases: MEDLINE (Ovid), MEDLINE-In Process (OVID), MEDLINE Epub Ahead of Print (Ovid), EMBASE (Ovid), Cochrane Database of Systematic Reviews (Cochrane) and Econlit (Ovid). Published HTA reports were identified through searching the International HTA Database (International Network of Agencies for Health Technology Assessment). Citation referencing and checking was performed on included studies. The search was conducted on June 13th and 14th, 2022.

The search strategy was structured based on the National Institute for Health and Care Excellence’s existing TB population search strategy (used in the 2016 NICE NG33 guidelines for the management of TB) and its most recent economic evaluation search strategies that were used in recently published systematic reviews (16–18). This strategy consisted of using terms related to TB (e.g., mycobacterium), disease pathology (e.g., phlegm), or existing disease diagnosis techniques (e.g., bronchoalveolar lavage), and pairing these search terms with existing terms related to current antitubercular agents (e.g., isoniazid). We then applied economic evaluation filters to these results. We limited results to articles published in English after January 1st, 2020.

The search for HTA reports was conducted in the International HTA database, utilizing a search string focused on TB or synonymous names for the disease. Further detail on all search strategies can be found in the Supplemental material.

### Eligibility criteria

Our review protocol specified that studies can be included if they were full economic evaluations (including cost-minimization, cost-utility, cost-effectiveness, cost–benefit, and cost-consequence studies) or cost-comparison studies evaluating two or more pharmaceutical interventions designed to treat one or more suspected or confirmed active variants of *mycobacterium tuberculosis*.

Studies were excluded based on the first criteria they met in the following order:

1. The target population did not have tuberculosis.
2. The intervention was a diagnostic technology, a preventive intervention (e.g., vaccines), or a public health intervention (e.g., face coverings, population screening).
3. The study was a partial economic evaluation.
4. The study was a modeling study that predicted epidemiological outcomes over time without an economic evaluation.
5. The study was a poster abstract that did not provide sufficient methodological detail.
6. The study was a letter to the editor, commentary, or editorial.

Results were first screened under the selection criteria by one reviewer (S.N.) based on titles and abstracts, with a second reviewer (D.D.) reviewing 10% of the previous sample to ensure screening was done correctly. Records that potentially met inclusion criteria were assessed in full. The decision to exclude studies after full-text review was done by both reviewers, with any remaining disagreements resolved through discussion. Data extraction was performed on included studies. Studies included in the Byun et al. review were reassessed using the same criteria, for inclusion in our review (15).

### Data extraction

Data extracted from the published studies included study characteristics, consisting of the country, currency, setting, interventions and comparators, type of economic evaluation, analysis approach, study perspective, model time horizon, cost categories, cost year, discounting rates, health outcomes, and sources of efficacy and utility data (see Table 1). Study results were also collected, consisting of cost and health outcome results, incremental cost-effectiveness ratios, net benefits of interventions versus, comparators, cost-effectiveness thresholds, sensitivity and scenario analysis, the authors’ conclusions regarding cost-effectiveness, and the authors’ reported limitations and challenges (see Table 2).

**Table 1:** Background details of included studies.

Study	Year	Country	Currency	Population/ Setting	Intervention(s) & comparator(s)	Type of evaluation	Analysis approach	Perspective	Time horizon	Cost categories	Cost year	Discounting
Manabe et al. (19)	2012	Uganda	USD	Both HIV positive and negative patients with active TB	isoniazid plus ethambutol for 6 months (6HE) vs. isoniazid + rifampicin for 4 months (4HR)	Cost-Effectiveness Analysis	decision tree model	Healthcare	Not reported	drug costs, clinic visit costs, retreatment costs	2008	Not reported
Law et al. (20)	2013	Ecuador	INT USD	100,000 smear-positive, treatment naïve patients	standard WHO regimen vs. ethambutol +6 month treatment regimen vs. strengthened standardized retreatment regimen vs. standardized MDR treatment	Cost-Utility Analysis	Markov model (7 states)	Societal	10 year	direct patient costs, indirect patient costs	2010	3%
Owens et al. (21)	2013	Multiple countries- not specified	USD	Hypothetical cohort of 100 individuals with active TB (confirmed using sputum culture or smear) with no known resistance	hypothetical drug with shorter duration, equal efficacy, and higher cost treatment regimen vs. standard regimen (isoniazid, rifampin, pyrazinamide, ethambutol) being treated in the public sector in an area of low background drug resistance or with known drug susceptibility.	Cost-Utility Analysis	Decision tree model	Healthcare	lifetime horizon	treatment costs (drug costs, delivery costs)	2012	3%
Wolfson et al. (22)	2015	UK	GBP	adult patients with pulmonary MDR-TB	bedaquiline + background regimen (BR) vs. BR alone. BR not specified.	Cost-Utility Analysis	Markov model (6 states)	Healthcare (NHS and PSS)	10 year	direct medical costs (drug acquisition costs, treatment monitoring costs, inpatient costs, outpatient costs, cost of surgical intervention).	2013	3.50%
Knight et al. (23)	2015	South Africa	USD	6 million patients predicted with TB disease between 2 years	4 month vs. 6 month regimen of therapy	Cost-Utility Analysis	individual-based simulation/transmission model	Societal	20 years	diagnostic costs, first-line treatment costs, MDR treatment costs, antiretroviral therapy costs	2015	3%
Diel et al. (24)	2015	Germany	EUR	MDR-TB patients	Delamanid + background regimen vs. BR regimen. Background regimen not specified.	Cost-Effectiveness and Cost-Utility Analysis	Markov Model (4 states)	Societal	10 years	drug costs, inpatient costs, outpatient costs	2015	3%
Park et al. (25)	2016	South Korea	KRW	patients with MDR-TB or XDR-TB	Bedaquiline + standard regimen vs. standard-regimen. Standard regimen was not specified.	Cost-Utility Analysis	Markov Model (9 states) (adaptation of Wolfson et al. model)	Healthcare	20 years	direct medical costs (drug acquisition costs, monitoring costs, inpatient costs, outpatient costs), transportation costs, care assistant costs	2014	5%
Gomez et al. (26)	2016	South Africa, Brazil, Bangladesh, Tanzania	USD	10,000 individuals with newly diagnosed pulmonary TB and no treatment history, all described countries	six-month regimen vs. four-month regimen	Cost-Utility Analysis	decision tree model	Healthcare, Societal	Unclear	healthcare provider costs (guideline), patient costs (guideline), healthcare provider costs (current), patient costs (current)	2013	3%
Codecasa et al. (27)	2017	Italy	EUR	all disease stages with MDR-TB and XDR-TB	Bedaquiline + background drug regimen (BBR) vs. background drug regimen (BR). Details of BR not mentioned.	Cost-Effectiveness and Cost-Utility Analysis	Markov Model (8 core health states for MDR-TB, 6 for XDR-TB patients)	Healthcare, Societal	10 year	drug costs, outpatient costs, end-of-life care costs, productivity costs, transmission costs	Unclear (2016 likely)	3%
Lu et al. (28)	2017	Estonia, Russia, South Africa, Peru, China, The Philippines, India	USD	laboratory confirmed cases of MDR-TB, all described countries	Bedaquiline + background drug regimen (BR) vs. background drug regimen (BR). BR was not specified.	Cost-Utility Analysis	Markov Model (11 states for MDR-TB, 6 states for XDR-TB) (adaptation of Wolfson et al. model)	Healthcare	10 year	direct medical costs, treatment monitoring costs, hospitalized costs, outpatient costs	2013	6% (China), 3% (Peru), 5% (Estonia, Russia, South Africa, India, Philippines)
Schnippel et al. (29)	2017	South Africa	USD	patients receiving ambulatory treatment in high-HIV prevalence setting	bedaquiline-based regimen vs. kanamycin-based regimen	Cost-Utility Analysis	Markov Model (12 states)	Provider	10 year	drug costs, laboratory testing costs, other investigation costs, care costs	2016	3%
Schnippel et al. (30)	2017	South Africa	USD	HIV positive patients with MDR-TB needing ambulatory treatment	bedaquiline + standard long-course treatment vs. second-line injectables (SLIs) + standard, long-course treatment	Cost-Utility Analysis	Markov Model (12 states)	Provider	10 year	TB drug/component costs, TB monitoring costs, adverse drug reaction (ADR) component costs, ADR management costs	2016	3%
Wirth et al. (31)	2017	Germany	EUR	100 patients with MDR-TB	bedaquiline + background regimen (BR) vs. delaminid + BR vs. linezolid+ BR vs. BR alone. BR not specified.	Cost-Effectiveness and Cost-Utility Analysis	Markov model (6 states)	Healthcare	10 year	direct medical costs (drug acquisition costs, treatment monitoring costs, administered care costs, end of life care costs, adverse events costs)	2015	3%
Fan et al. (32)	2019	Hong Kong	USD	Hypothetical cohort of adult patients with MDR-TB	bedaquiline + background regimen vs. delamanid + background regimen vs. background regiment alone. BR not specified.	Cost-Utility Analysis	decision tree model	Provider	10 year	direct medical costs (drug costs, hospitalization costs, follow-up costs)	2017	3%
Manalan et al. (33)	2020	UK	GBP	100 known patients treated with injectables	amikacin vs. bedaquiline	Cost-Comparison Analysis	Retrospective analysis	Healthcare	treatment duration (up to 8 months)	drug costs	2019	No discounting
Agnarson et al. (34)	2020	South Africa	USD	simulated MDR-TB cohort	bedaquiline-containing short course regimen vs. injectable-containing short course regimen	Cost-Utility Analysis	Markov Model (8 states)	not reported	10 year	outpatient costs, inpatient costs, monitoring costs, adverse events cost, productivity cost	2019	5%
Madan et al. (35)	2020	Ethiopia, South Africa	USD	119 individuals in Ethiopia, 47 individuals in South Africa	long vs. short TB treatment regimens	Cost-Effectiveness Analysis	economic analysis alongside clinical trial	Healthcare, Participant	132 weeks	Drug costs, inpatient stays costs, adverse events costs, laboratory testing costs electrocardiography costs, staff time costs, consumables costs, social support costs.	2017	Not reported
Reddy at al. (36)	2020	South Africa	USD	patients with TB and HIV co-infection	Novel 4 month regimen vs. standard 6 month regimen	Cost-Effectiveness Analysis	Monte Carlo microsimulation model (Cost-Effectiveness of Preventing AIDS Complications International)	Healthcare	lifetime horizon	drug costs, follow-up costs, laboratory monitoring costs	2017	3%
Bada et al. (37)	2020	Nigeria	USD	DR-TB susceptible individuals	3 shorter treatment regimens currently used in Nigeria vs. 3 longer ones not currently used.	Cost-Comparison Analysis	Comparison of treatment regimens	Healthcare	9 month	diagnostic costs, monitoring test costs, drug costs, inpatient costs, follow-up testing costs	2020	NA
Gomez et al. (38)	2021	South Africa, Georgia, the Philippines	USD	patients with XDR-TB	Bedaquiline, pretomanid and linezolid (BPaL) regimen vs. local standard of care. Local standard of care not specified.	Cost-Utility Analysis	Markov Model (8 states)	Healthcare	lifetime horizon	drug costs, testing costs, monitoring costs, palliative care costs, antiretroviral treatment costs	2018	3%

DALYs, Disability-adjusted life-years; GBP, Great Britain Pound; NA, Not applicable; QALY, Quality-adjusted life-year; TB, Tuberculosis; USD, United States Dollars.

**Table 2:** Summary of the results of included studies.

Study	Primary health outcomes (mean per patient)	costs (mean per patient)	ICER/net benefit of intervention(s) vs. comparator(s)	Cost-effectiveness threshold (if relevant)	Sensitivity & scenario analyses	Authors’ conclusions regarding cost effectiveness	Authors’ reported limitations and challenges	value considerations discussed
Manabe et al. (19)	Model 1: 6HE: 13.3% mortality rate 4HRL 8.8% mortality rate.	Model 1: 6HE: $12.77. 4HRL $13.66.	4HR was dominant over 6HE in both models	Not applicable	4HR widely dominated 6HE in a wide range of sensitivity analyses	“A transition to the strongly recommended continuation phase 4HR regimen is associated with lower costs, lower mortality, a lower overall risk for relapse and, therefore, a reduced need for retreatment.”	Cost estimates from perspective of Ugandan health system, with costs from local rates, Analysis based on clinical efficacy results obtained in RCTs/cohort studies, with different actual efficacy Most studies not in Rwanda Did not evaluate impact of increasing MDR-TB rates. GLC MDR treatment may be more effective, but drugs not available and allow for different treatment in the national system.	
Law et al. (20)	Outcomes (per 100,000 patients): 5% INH monoresistant TB, 1% MDR-TB standard: reference, emb initial: −641 (−660–-622) DALYs str retreat: 91 (90–92) DALYs, mdr failures: 613 (609–617) DALYs. 15% INH monoresistant, TB 1% standard: reference, emb initial: −237 (−258–-216) DALYs str retreat: 191 (189–193) DALYs, mdr failures: 889 (878–900) DALYs 5% INH monoresistant, TB 10%: standard: reference, emb initial: −660 (−679–-641) DALYs str retreat: 91 (90–92) DALYs, mdr failures: 5177 (5157–5,197) DALYs 15% INH monoresistant, TB 10%: standard: reference, emb initial: −255 (−276–-234) DALYs str retreat: 192 (191–193) DALYs, mdr failures: 5454 (5432–5,476) DALYs	Costs: 5% INH monoresistant, TB 1%: standard: 4,697 Int $, emb initial: 4,687 Int $, str retreat: 4,697 Int $, mdr failures: 4,732 Int $. Costs: 15% INH monoresistant, TB 1%: standard: 4,771 Int $, emb: 4,756 Int $, str retreat: 4,763 Int $, mdr failures: 4,814 Int $. Costs: 5% INH monoresistant, TB 10%: standard: 5,009 Int $, emb: 5,000 Int $, str retreat: 5,009 Int $, mdr failures: 5, 157 Int $. Costs: 15% INH monoresistant, TB 10%: standard: 5,084 Int $, emb: 5,069 Int $, str retreat: 5,075 Int $, mdr failures: 5,240 Int $.	5% INH monoresistant, TB 1%: standard vs. EMB — less effective, str retreat vs. standard — dominant, MDr failures vs. standard —Int $5,745/DALY. 15% INH monoresistant, TB 1%: standard vs. EMB — less effective, str retreat vs. standard — dominant, MDr failures vs. standard — Int $4,867/DALY 5% INH monoresistant, TB 10%: standard vs. EMB — less effective, str retreat vs. standard — dominant, MDr failures vs. standard —Int $2,857/DALY 15% INH monoresistant, TB 10%: standard vs. EMB — less effective, str retreat vs. standard — dominant, MDr failures vs. standard — Int $2,860/DALY	1 GDP *per Capita*	EMB was least likely to be cost-effective. MDR failures become more likely to be cost-effectiveness than both strengthened retreatment and EMB initial as WTP threshold increases. But strengthened retreatment is more cost-effective below certain thresholds.	Strengthened retreatment regimen can boost cost savings and increase treatment effectiveness.	Disease transmission was not modeled. Longer duration of disease causes lower quality of life, which may have implicated DALYs. Impact of treatment outcomes is limited by limited evidence and based on studies in Ecuador. HIV not explicitly considered in model either.	Co-infection, empirical treatment
Owens et al. (21)	Standard treatment: 1.35 DALYs. 4-month regimen: 1.27 DALYs. 2 month: 1.21 DALYs	Standard treatment: low-cost: 126 USD, moderate costs: 226 USD, high cost: 985 USD costs. 4-month regimen: low-cost: 184 USD, moderate cost: 260 USD, high cost: 832 USD. 2 month: low cost: 32800 USD, moderate cost: 377 USD, high cost: 753 USD.	4 month vs. standard: low-cost: 740 USD/DALY, moderate costs: 430 USD/DALY, high costs: Preferred (dominant). 2-month vs. standard: low-cost: 1400 USD/DALY, moderate cost: 1000 USD/DALY, high-cost: preferred (dominant).	Less than 1 per capita gross domestic product (GDP) being ‘highly cost-effective,’ and less than 3 times *per capita* GDP being ‘cost-effective	Three primary drivers of cost-effectiveness: Drug costs Cost of treatment delivery in continuation phase Ability of novel regimens to prevent death in episodes of recurrent TB.	“novel regimens for the treatment of drug-susceptible TB in the public sector are likely to be cost-effective or cost-saving in the majority of economic and epidemiological conditions.”	Simplified model based on WHO-estimates and arbitrary thresholds — based on continuing states.	adherence to treatment, treatment delivery costs
Wolfson et al. (22)	Bedaquiline + BR: 5.16 QALYs, BR: 4.01 QALYs	Bedaquiline + BR: £106,487 BR: £117,922	Dominates (−10,008.75 GBP/QALY gained)	20,000–30,000 GBP/QALY	PSA: “The probability that bedaquiline plus BR is cost-effective versus BR alone at an affordability threshold of £20,000 per QALY gained (30) and £50,000 per QALY gained (22) was 96 and 99%, respectively. The strategy of bedaquiline plus BR was cost-saving (and dominant) versus BR alone in 81% of probabilistic simulations.”	Bedaquiline is 81% certain to produce cost-savings if sold at +20/−20% of US list price and would lead to improved qualityof life. Bedaquiline _ BR is dominant (less costly + more effective) with standard of care.	Model does not capture mortality imbalances from C208 Model’s source of data is a small Phase II placebo-controlled trial outside of UK, may not be representative Patients lost to follow-up were assumed to do so until death Model assumes that sputum culture conversion saw no more disability or death.	orphan indication, inclusion of transmission dynamics
Knight et al. (23)	Current: Standard — 5.45 DALYs, New — 5.48 DALYs. (other data reported for policy and guideline scenarios)	Current (calculated from reported data): Standard — 62.4 USD New — 47.62 USD.	Four-month regimen at which cost per DALY averted equated threshold: $436 [NA, 5983]	6,618 USD (WTP is DP *per capita*)	Scenario analysis: Impact of the 4-month regimen was similar in all explored scenario, with a less than 3% change.	New four-month regimens are highly likely to be cost-effective in South Africa.	Model has uncertainty issues – did not include the most recent antiretroviral therapy (ART) — over predict levels of HIV and hence TB disease. Characteristics of TB status depended on HIV status but not immune analysis. Did not consider indirect costs past 20-year time horizon. Did not include regimen costs. Did not include economic effects of resistance.	
Diel et al. (24)	Patients with Deltyba 8.47 QALYs gained. Patients with BR: 6.13 QALYs gained,	Patients on Deltyba: 142,732 Euros, 157005.2 USD. Patients with BR: 15090 Euros; 16,599 USD.	Dominates (−3,494 EUR; 3842.83 USD)	WTP threshold is 10,000	Deterministic and probabilistic sensitivity values were conducted; found sensitivity to cost changes	Deltyba added to background regimen is likely to be cost-effective	Model solely from patients outside Germany Did not capture culture conversion costs Patients who were once lost to follow-up remained lost over horizon and did not get treatment	productivity gain
Park et al. (25)	Experimental: 5.20 QALYs, Comparator: 3.80 QALYs, Incremental: 1.20 QALYs,	Experimental: 86,043,831 KRW Comparator: 72,082,172 KRW Incremental: 13,961,659 KRW	incremental cost/utility ratio: 11,638,656 KRW/QALY incremental cost-effectiveness ratio: 10,822,992 KRW/life year gained	26 million KRW	PSA and DSA. The values of 10 parameters varied at around +/−20% in the 1-way DSA, and the results were generally stable	Study concludes bedaquiline + SR is cost effective in comparison to SR alone with a probability of 80% at the specified threshold	C208 study for data did not include XDR-TB, and XDR-TB data were derived from the hazard ratio of a different, single-arm study. Limited data on utility weights of MDR-TB Utility weights were only based on one study from Thailand (and was not country specific). Transition probabilistic for sputum culture conversions from one data source.	
Gomez et al. (26)	Guidelines: South Africa: 6 month: 9.97 DALYs averted. 4 month: 10.0 DALYs averted. Brazil: 6 month: 16.50 DALYs averted. 4 month: 16.54 DALYs averted. Bangladesh: 6 month: 16.19 DALYs averted. 4 month: 16.22 DALYs averted. Tanzania: 6 month: 13.66 DALYs averted. 4 month: 13.67 DALYs averted. Current: South Africa: 6 month: 8.26 DALYs averted. 4 month: 8.37 DALYs averted. Brazil: 6 month: 14.68 DALYs averted. 4 month: 15.18 DALYs averted. Bangladesh: 6 month: 16.17 DALYs averted. 4 month: 16.20 DALYs averted. Tanzania: 6 month: 12.97 DALYs averted. 4 month: 13.00 DALYs averted.	Healthcare costs: Guidelines: South Africa: 6 month: 1165.5 USD. 4 month: 1145.4 USD. Brazil: 6 month: 1972.5 USD. 4 month: 1509.1 USD. Bangladesh: 6 month: 125.7 USD. 4 month: 200.6 USD. Tanzania: 6 month: 222.5 USD. 4 month:294.7 USD. Current: South Africa: 6 month: 563 USD. 4 month: 610.4 USD. Brazil: 6 month: 950.2 USD. 4 month: 790.3 USD. Bangladesh: 6 month: 109.2 USD. 4 month: 184.4 USD. Tanzania: 6 month: 152.8 USD. 4 month:223.8 USD.	Guidelines: South Africa: ICER: cost-saving. Brazil: ICER: cost-saving. Bangladesh ICER: USD 164. Tanzania: ICER: cost-saving. Current: South Africa: ICER: USD 16.9. Brazil: ICER: cost-saving. Bangladesh: ICER: USD 129. Tanzania: ICER: USD 39	WTP of quarter, half or 1 times GDP *per capita*. GDP *per capita* – South Africa: USD 6,618, Brazil USD 11,208, Bangladesh: USD 829, Tanzania: USD695	One-way sensitivity analysis: conclusions stable to assumption, with cost-effectiveness most sensitive to existing health service costs for delivery and default rates. Higher MDR mean regimen was more cost saving in Brazil/South Africa, while Bangladesh has regimen being cost-saving, and regimen is cost-effectiveness in Tanzania under all analyses.	“A four-month non-inferior first-line TB regimen is likely to be cost saving or cost-effective in many country settings.” Benefit is larger in middle income countries, and drug price is more critical in low-income countries.	Excluded benefits to children Excludes benefits in prevention of downstream transmission Excludes benefits in prevention of acquired resistance. Did not include program cost or the influence of alternate approaches.	transmission, resistance
Codecasa et al. (27)	BBR: 5.18 LYGs (NHS), 5.18 (Societal). BR: 51,615, 5.18 LYGs (NHS). 85,875, 4.17 (Societal).	BBR: EUR 68323 (NHS), EUR 89973 (Societal). BR: EUR 51615, (NHS) EUR 85875 (Societal).	Incremental: 15684/QALY (NHS), 3,847 (Societal), 4.36 (BBR) vs. 3.29 (BR) NHS perspective: BBR dominant in 19% of cases. Societal perspective: BBR dominant in 45% of cases.	40,000 EUR/LYG - 60,000 EUR/LYG	PSA: BBR is almost certainly cost-effective vs. BR (in 88 and 96% of cases)	BBR vs. BR is a cost-effective strategy and is far more cost-effective from the societal perspective.	Analysis was developed using assumptions that simplified treatment pathway There is no efficacy data on BBR versus BR alone in patients with XDR-TB, with an MDR-TB study used for data instead. Weaknesses in data for either comparator.	productivity gain
Lu et al. (28)	Experimental: DALYs per patient: Estonia: 11.54. Russia: 11.14. South Africa: 12.14. Peru: 14.75. China: 8.87. Philippines: 13.86. India: 14.43. Comparator: Estonia: 14.59. Russia: 13.84. South Africa: 14.86. Peru: 18.57. China: 11.86. Philippines: 16.15. India: 18.54 Incremental: Estonia: −20.90. Russia: −19.51. South Africa: −18.35. Peru: −20.59. China: −25.15. Philippines: −14.16. India: −22.18	In USD, excluding drug acquisition costs, Estonia: $33,202. Russia: $29,615. South Africa: $6,667. Peru: $7,337. China: $4,201. Philippines: $1,528. India: $201.	No ICER reported. A price threshold analysis showed that in Estonia, Russia, Peru, and China the price range that results in cost effectiveness at a threshold of 3 GDP *per capita* ranged between US$23,904-US$203,492. The range for South Africa was lower at US$29,151-US$72,701, while the Philippines and India demonstrated a lower range, at US$6,996-US$20,323	1 and 3 x GDP *per capita* (Estonia, Russia, China, Peru: $23,904–$203492’South Africa: $29,151–$72,701; Philippines/India: $6,996 - $20,323)	PSA. Additionally, A sensitivity analysis evaluating the outcomes of treatment with bedaquiline in a cohort of XDR-TB patients only, demonstrated that bedaquiline was associated with greater DALYs averted in this patient group. When transmission rates were included in sensitivity analyses, bedaquiline was associated with additional healthcare cost savings associated with the reduced number of cases (data not shown).	BBR improves health outcomes with a reduced DALY burden compared with a BR alone. BBR is 32–94% cost-effective in the burdens provided.	clinical data on the phase II study for bedaquiline is multinational and does not reflect local data. Study also used UK life tables to calculate DALYs since country-specific DALYs were not possible. Limitations also include that possible increases in mortality due to the bedaquiline has been excluded, as well as a lack of empirical data.	transmission, value of innovation, budget impact
Schnippel et al. (29)	MDR/RR-TB standard regimen: 5.12 DALYs. MDR/RR-TB standard regimen + bedaquline for XDR-TB: 5.14 DALYs. MDR/RR-TB bedaquiline for all: 5.29 DALYs.	MDR/RR-TB standard regimen: 4,439 USD. MDR/RR-TB standard regimen + bedaquline for XDR-TB: 4,356 USD. MDR/RR-TB bedaquiline for all: 4,648 USD.	The incremental cost-effectiveness ratio (ICER) of bedaquiline for all MDR/RR-TB was $US1242 per additional DALY averted compared with the standard regimen. The incremental cost-effectiveness ratio (ICER) of bedaquiline for XDR/RR-TB was $US3804 per additional DALY averted compared with the standard regimen.	2015 *per capita* GDP for South Africa of $US5718	Deterministic sensitivity analysis: “Overall, the analysis was most sensitive to the proportion of patients who culture converted while receiving the standard regimen: a 25% increase in culture conversion on standard regimens led to a 2.3-fold increase in the ICER for bedaquiline for all ($US3908 per DALY averted). Results were also sensitive to a 25% increase in the cost of bedaquiline, which led to a 90% increase in the ICER ($US2242 per DALY).”	Bedaquiline for all patients increased treatment success-rate and was cost-effective. Standard regimens without bedaquiline were dominated by other regimens.	Did not use data from the phase IIb trial for mortality due to imbalance. Did not include a measure of ongoing transmission Perspective does not reflect societal costs.	budget impact
Schnippel et al. (30)	No toxicity profile: injection-based: 4.88 DALYs. BDQ-based: 4.64 DALYs. Adjusted for toxicity: injection-based: 5.60 DALYs. BDQ-based: 4.64 DALYs	No toxicity profile: injection-based: 4614 USD. BDQ-based: 4739 USD. Adjusted for toxicity: injection-based: 4996 USD. BDQ-based: 4899 USD.	ICER: No toxicity profile: injection-based vs. BDQ-based: 0.24 DALYs, 124 USD Adjusted for toxicity: injection-based vs. BDQ-based: 0.96 DALYs, 4,899 USD. (the reference in the toxicity-adjusted case was dominated). The BDQ regimen absolutely dominated the SLI regimen, saving US$96 and averting 0.96 DALYs per patient over the modeled period.	Threshold not specified	Probabilistic uncertainty analysis: showed that 80% of distributions had an ICER of less than $2,100/DALY, and when toxicity adjusted, 80% of ICER was below $150/DALY and 62% was cost-saving and more effective (dominant).	Current treatments can have high rates of ADR (adverse events), and new drugs may be more cost saving and more effective if adverse events are accounted for, with bedaquiline being one example of a new drug.	Same mortality rates were used as for standard regimens, not relying on Phase iiB findings which had bias. Did not include a measure of ongoing transmission Did not increase costs due to ADRs Excluded patients’ direct and opportunity costs	Adverse events
Wirth et al. (31)	BR: 3.68 QALYs. L inezolid + BR: 3.91 QALYs. Delamanid + BR: 5.36 QALYs. Bedaquiline + BR: 5.95 QALYs	BR: €60,962 EUR. Linezolid + BR: €80,460 EUR. Delamanid + BR: €81,079. Bedaquiline + BR: €85,575.	BR: Comparator. Lenazolide +BR: dominated. Delamanid +BR: dominated. Bedaquiline + BR: 22,238 €/QALY.	30,000–50,000 €/QALY	PSA: the probability of being the most cost effective strategy for Bedaquiline + BR was 54.5%, higher than 22.9% for BR alone, 4.4% for linezolid + BR, and 18.2% for delamanid + BR	“The addition of bedaquiline, delamanid, or linezolid to a BR would result in QALY gains over BR alone when applied in the German healthcare system. Bedaquiline is likely to be the most cost-effective intervention for the treatment of MDR-TB, when added to a BR regimen at thresholds greater than €22,000 per QALY.”	Bedaquiline + delamanid may not accurately reflect German clinical practice Mortality imbalance in C208 trial was not accounted for Patients lost to follow-up were assumed to be lost-until death Patients with sputum culture conversion did not have disability Heterogeneity between studies introduces uncertainty	treatment duration, adverse events, development of resistance, route of administration
Fan et al. (32)	BR: 6.347 QALYs (reference). Bedaquiline + BR: 7.078 QALYs (Incremental QALYs 0.731 QALYs). Delamanid + BR: 6.359 QALYs (Incremental QALYs 0.012)	BR: 47,396 USD (reference). Bedaquiline + BR: 47405 USD (incremental cost 9 USD) Delamanid + BR: 67560 USDs (incremental cost 20,164 USD)	ICER: Bedaquiline + BR vs. BR: 12 USD/QALY. Delamaind + BR: 1,680,333 USD/QALY	46,182 USD (1 times GDP *per capita*)	PSA: “as the WTP threshold, B-BR and D-BR were cost-effective 99.98 and 5.13% of the time, respectively.”	Bedaquiline BR is cost-effective with an ICER below WTP, while add-on delamanid + BR is unlikely to be cost-effective	Sources of outcome events data was simulated with oversees data, requiring extended ranges in sensitivity analysis. Model also simplified MDR-TB outcomes, so total cost may be underestimated	
Manalan et al. (33)	NA	Mean cost: observed injectable: 2,723.6 GBP. Amikacin: 6 month: 3,026.4 GBP 8 month: 2,176.0 GBP. Bedaquiline: 3,176.0 GBP.	NA	NA	None reported	Bedaquiline is cost neutral as compared to treatment with an injectable.	Amikacin costs may be underestimated. Pricing of bedaquiline and potential drop in prices not accounted for.	Adverse events
Agnarson et al. (34)	Total DALYs: bedaquiline-SCR: 734,536. injectable SCR: 793,556.	Total costs: bedaquiline SCR: 596,538,583 USD. Injectable: 657,212,525 USD.	Incremental DALYs: bedaquiline SCR vs. injectable SCR: −61,805. Incremental costs: −60,673,941. ICER: 982 USD saved per DALY averted.	6,160 USD/DALY averted (based on GDP *per capita*)	Deterministic Sensitivity Analyses: “the bedaquiline-containing SCR demonstrated cost savings and prevented more DALYs compared with the injectable-containing SCR”	Bedaquiline SCR cost effective against injectable SCR in South Africa	Memory-less transition states of Markov Model means history of cohort not captured. Absence of patient-level data between both regimens means relative risk was used for culture conversion. Treatment costs may be higher due to high prevalence of HIV-positive individuals Cost of hospitalization was from a single hospital rather than national.	adverse events, productivity
Madan et al. (35)	Not reported	Health-system costs. Ethiopia. Long: 6,096.6 USD per participant. Short: 4,552.3 USD per participant. South Africa: Long: 8,340.7 USD per participant. Short: 6,618.0 USD per participant. Participant costs: Ethiopia: Long: 575.4 USD. Short: 337.3 USD.	Not reported	Willingness-to-pay thresholds up to US$ 100,000 for both Ethiopia and South Africa	PSA and bootstrapping: Short-regimen was highly likely to be cost-effective	Short-regimen of MDR treatment led to substantial savings for participants and healthcare system.	Cannot assert short-regimen is cost-effective because precise value on avoiding unfavorable otcomes is not specified. Could not estimate cost of adverse events in South-Africa. Missing data present, but sensitivity analysis showed little change if found. Did not include costs or consequences or treatment failure. participant costs were only calculated for Ethiopia.	productivity gain, catastrophic expenditure, drug resistance
Reddy at al. (36)	Model-generated outcomes: 6 m: 14.2 yr. life expectancy 4 m: 14.0 yr. life expectancy.	Model-generated Cost: 6 m: 9,090 USD. 4 m: 9,120 USD.	ICER: 6 m was dominant over 4 m.	USD 940/LYG and USD500/YLS and USD2,000/YLS.	PSA and one way sensitivity analyses undertaken to identify scenarios where the 4 m regimen would have more favorable cost effectiveness	Novel 4 m regimen could be cost-effective relative to 6 month regimen under certain assumptions that takes into account the importance of loss to follow up	Calibrated base model to trial data and assumed that monthly probability of loss to follow-up, and did not assume it would change. Did not model transmissions. Did not include costs or savings to patients. No country-specific preference weights were in the model.	loss to follow-up, HIV related complications
Bada et al. (37)	NA	Total cost: Model A: 14781.16 USD. Model B: 12,112.78 USD. Model C: 7,572.14 USD. Model D: 4,333.85 USD. Model E: 7,705.07 USD. Model F: 3,419.58 USD	NA	NA	NA	Model F is recommended choice if improved outcomes using bedaquiline-treated shorter regimen is used.	83.5% inflation since the cost of bed day. Cost of line probe assay was first-line alone. Cost of managing adverse drug reactions not counted Did not capture patient costs which contribute significantly to cost of managing RR/MDR-TB.	
Gomez et al. (38)	Total DALYs: South Africa: XDR-TB Cohort: SoC: 14,007 DALYs. BPaL: 14,007 DALYs. XDR-TB and MDR-TB intolerant/failure Cohort: SoC: 33,115 DALYs. BPaL: 17,699 DALYs. Georgia: XDR-TB Cohort: SoC: 893 DALYs. BPaL: 396 DALYs. XDR-TB and MDR-TB intolerant/failure Cohort: SoC: 1,491 DALYs. BPaL: 661 DALYs. The Philippines: XDR-TB Cohort: SoC: 268 DALYs, BPaL: 119 DALYs. XDR-TB and MDR-TB intolerant/failure Cohort: SoC: 11,890 DALYs. BPaL: 5,316 DALYs. Results reported for the whole cohort. Cohort size not reported.	Total TB related costs: South Africa: XDR-TB Cohort: SoC: 5,206,829 USD. BPaL: 5,206,829 USD. XDR-TB and MDR-TB intolerant/failure Cohort: SoC: 12,378,747 USD. BPaL: 4,414,849 USD. Georgia: XDR-TB Cohort: SoC: 282,680 USD. BPaL: 83,775 USD. XDR-TB and MDR-TB intolerant/failure Cohort: SoC: 478,439 USD. BPaL: 141,489 USD. The Philippines —XDR-TB Cohort — SoC: 84,237 USD. BPaL: 2,6,357 USD. XDR-TB and MDR-TB intolerant/failure Cohort — SoC: 3704919 USD. BPaL: 1,158,821 USD. Results reported for the whole cohort. Cohort size not reported.	NA (intervention cost saving in all tested comparisons)	Not reported	PSA: The potential threshold price at which the probability of BPaL becoming cost-neutral begins to increase is higher in Georgia and the Philippines as compared to South Africa	BPaL for treatment of XDR-TB is likely to be cost-saving at the proposed price.	Study was based on efficacy estimates from a small study without a randomized control. Linezolid and bedaquiline were used as part of standard of care (in both groups). However, newer trials confirm this data. Cost parameter values were estimated from guidelines and verified against empirical estimates, but were found to be lower, so cost-savings might be conservative.	transmission (not included), training, changes in guidance and changes in systems

DALYs, Disability-adjusted life-years; GBP, Great Britain Pound; GDP, Gross domestic product; NA, Not applicable; QALY, Quality-adjusted life-year; TB, Tuberculosis; USD, United States Dollar; WHO, World Health Organization.

### Quality assessment

We also assessed the quality of all included studies using the methodological limitations checklist provided by the NICE guidelines manual (39). Studies were evaluated to have either minor, potentially serious, or very serious limitations based on study characteristics, including sources of outcomes data, study assumptions, whether incremental cost-effectiveness ratios (ICERs) were reported, the relative rigor of sensitivity and scenario analyses. Completed methodological quality assessment of all included studies can be found in the Supplemental material.

Data extracted from the HTA reports included the intervention and comparator evaluated, the value elements discussed in each HTA report, and the final recommendation on the cost-effectiveness and value of the appraised intervention.

## Results

### Search results

**Figure 1:**
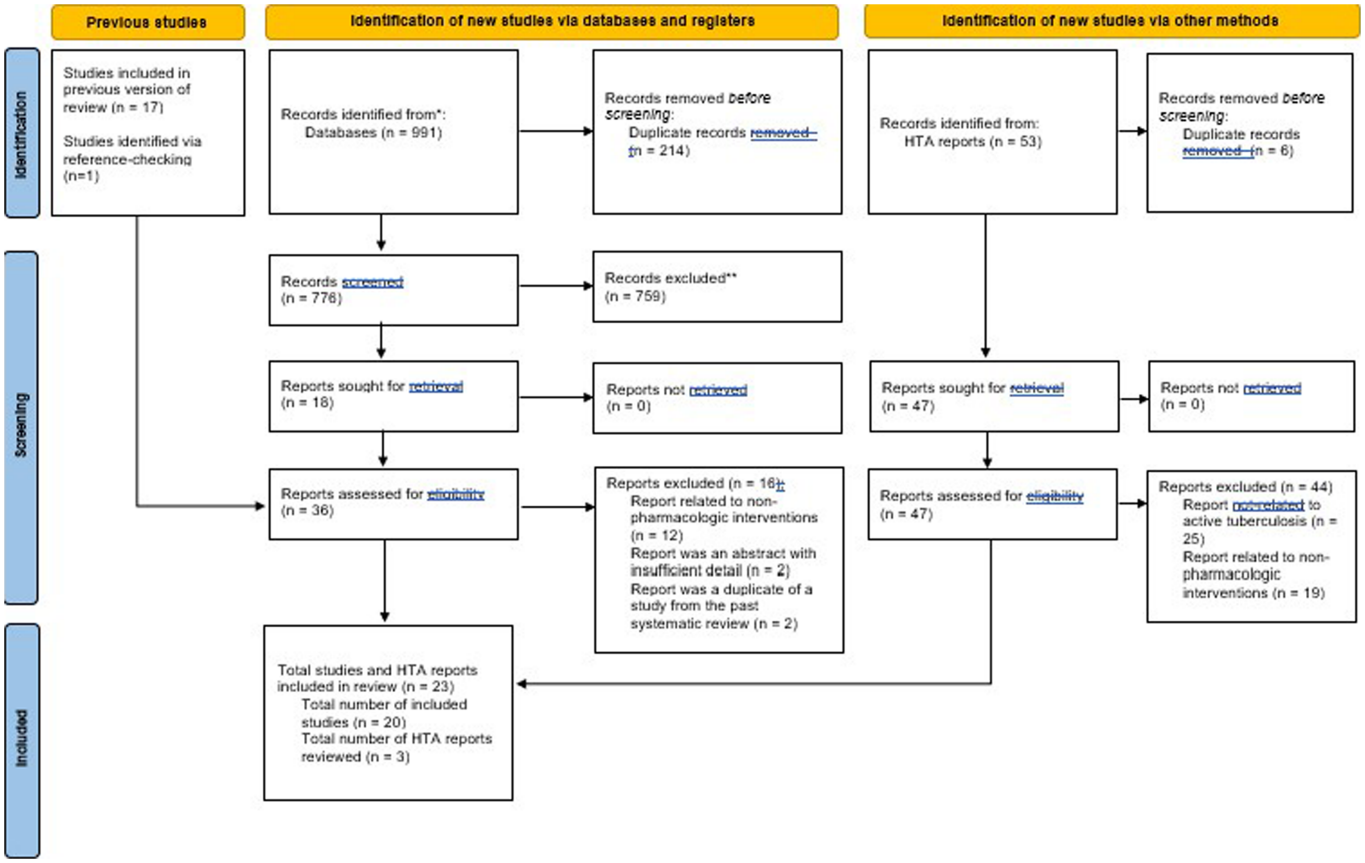
PRISMA 2020 flow diagram for updated systematic reviews which included searches of databases, registers and other.

Our search returned 991 published studies, which was combined with the 17 previous studies identified from the past systematic review by Byun et al. (15). After screening, 20 total studies were included in the review, as described in the PRISMA flowchart (see Figure 1). The most common reason for exclusion of published studies was that studies were either not focused specifically on active TB or were not an economic evaluation.

A total of 53 HTA reports was found from our search of the INAHTA database, of which 50 were excluded, most commonly because evaluations focused on latent tuberculosis or non-pharmacological interventions. Therefore, 20 published studies were considered potentially includable in our review (19–38). Of the 20 studies, 4 studies were found to have minor limitations, while 14 studies were found to have potentially serious limitations, and 2 studies were found to have very serious limitations (19–38). Methodological quality limitations commonly found in studies included short time horizons, omission of relevant outcomes, or suboptimal sources of outcome and intervention effects data. The characteristics of the included studies are summarized in Table 1.

### Study characteristics

Sixteen studies out of 20 conducted cost-utility analyses, while 6 conducted cost-effectiveness analyses and 2 conducted cost-comparison analyses, while some studies conducted multiple analyses. Twelve studies used a multi-state Markov model to simulate TB infection and recovery, while 4 studies used a decision tree model that combined various outcomes weighted based on probabilities to estimate cost-effectiveness. Two studies reported economic evaluations conducted alongside randomized control trials (RCTs). Nineteen out of 20 studies did not specify a specific patient population, while 2 studies focused on patients receiving ambulatory treatment.

Ten out of 20 studies specifically focused on MDR-TB or XDR-TB while 11 remaining studied all forms of active TB. Studies were conducted across a large variety of international settings, with South Africa being the most common setting (7 out of 20). Most studies occurred in the year 2017 (5 out of 20) and results were reported in USD ($) (15 out of 20). Most studies also adopted a healthcare perspective (13 out of 20) as the sole or main perspective, while a smaller portion of studies only used a societal perspective (6 out of 20).

Of the 3 HTA reports evaluated, two reports evaluated the value of bedaquiline relative to background regimens (40, 41). The third HTA report by EUnetHTA evaluated the combination of a pretomanid, bedaquiline, and linezolid (BPaL) regimen against three comparator regimens of various common tuberculosis medications (42). Due to heterogeneity in the content of HTA reports, HTA results were not included in Tables 1, 2 (40–42).

Given the extensive heterogeneity and significant limitations posed when attempting quantitative synthesis of cost-effectiveness estimates, we conducted a qualitative, narrative synthesis of included studies. See Table 2 for summary of studies’ results.

### Cost-effectiveness of bedaquiline

Eleven of 20 studies evaluated the cost-effectiveness of bedaquiline versus a background regimen (BR) or other standard-of-care regimens. Codecasa et al. found that addition of bedaquiline to background regimen (BBR) in an Italian setting was cost-effective in 88 and 96% of cases, respectively, at thresholds of 40,000 EUR/life-year gained (LYG) and 60,000 EUR/LYG, with BBR having an ICER of 15,684 EUR/quality-adjusted life year (QALY) from a healthcare perspective and 3,847 EUR/QALY from a societal perspective (27). Park et al. similarly found that addition of bedaquiline to a standard regimen in a Korean setting was cost-effective in a majority of cases at a threshold of 26 million KRW with an ICER of 10,822,992 KRW/LYG (25). Wolfson similarly found in a British setting that addition of bedaquiline to a background regimen dominated a background regimen at thresholds of 20,000 and 30,000 GBP/QALY, with an ICER of 10,0008.75 GBP/QALY gained (22).

Schippel et al. drew similar conclusions South African setting, finding that adding bedaquiline to a standard regimen have an ICER of $1,242/disability-adjusted life-year (DALY) for patients with MDR/RR-TB, making it cost-effective at a threshold of 1 GDP (gross domestic product) *per capita* equal to $5,718 (29). A follow-up analysis in Schippel et al. also found that the cost-effectiveness of adding bedaquiline to existing regimens increased if the effects of adverse reactions to pharmacological treatments were included in cost-effectiveness determinations (30). Fan et al. agreed with this analysis in a Hong Kong setting, finding bedaquiline’s addition to a background regimen had an ICER of $12/QALY and was cost-effective in 99.98% of cases at a threshold of one GDP *per capita* equal to $46,182 (32). Agnarson et al. similarly found that bedaquiline was cost-effective over a generic injectable short-course regimen with an ICER of $982 USD/DALY at a threshold of $6,160 (34).

Other studies found similar results even when comparing the addition of bedaquiline to background regimen against other tuberculosis drugs. Wirth et al. found that, in a German setting, a bedaquiline plus background regimen strategy dominated both a linezolid plus background regimen and bedaquiline plus background regimen strategy with an ICER of 22,238 EUR/QALY gained at thresholds of both 30,000 and 50,000 EUR/QALY gained (31). Gomez et al. conducted a cost-comparison analysis in a six-country setting and similarly estimated that the addition of bedaquiline to a regimen of pretomanid and linezolid was most likely to be cost-effective compared to local standards of care (38). More pessimistically, a cost comparison analysis by Manalan et al. in a UK setting found bedaquiline to be cost-neutral compared to current injectable regimens (33).

Only one study, Lu et al., found mixed evidence on the cost-effectiveness of bedaquiline, finding adding the drug to standard regimens to be cost-effective in 32–94% of cases across four international settings (Estonia, China, Russia, and Peru) depending on if a threshold of one or three times GDP *per capita* was used (28). Lu et al. did not calculate an ICER for bedaquiline (28).

### Cost-effectiveness of shorter vs. longer duration regimens

Six studies evaluated the cost-effectiveness of using short-course regimens versus long-course regimens for the treatment of MDR-TB and XDR-TB. Knight et al. found that in a South African setting, a shorter 4 month-regimen was likely to be cost-effective over a 6-month regimen with an ICER of $436/DALY at a threshold of $6,618 (23). Gomez et al. also found a shorter regimen to be cost-effective across several settings at a threshold of one GDP *per capita* in each country (26). Cost-comparison analysis found similar results as well, with both Bada et al., in a Nigerian setting, and Madan et al. in an Ethiopian and South African setting finding that short-course treatment regimens were likely more cost-effective than longer regimens (35, 37). This result also aligned with the hypothetical modeling in Owens et al., which analyzed a hypothetical shorter-duration, higher-cost treatment in a hypothetical country setting and found the shorter regimen to be cost-effective as well (21). Only Reddy et al. contradicted these findings in a South African setting and found a 6-month regimen dominated a 4-month regimen at a $940/LYG threshold (36).

### Cost-effectiveness of ethambutol

Two studies assessed the cost-effectiveness of ethambutol as compared to other interventions. Law et al. found that ethambutol, when added to a standard WHO regimen, was least likely to be cost-effective at threshold of one GDP *per capita* in an Ecuadorian setting (20). Manabe et al. found that a 4-month regimen of isoniazid plus rifampicin dominated a 6-month regimen of isoniazid plus ethambutol by comparing mortality rates and costs of treatment (19). Both results suggest than ethambutol may not be a cost-effective addition to most standard TB treatment regimens.

### Cost-effectiveness of delamanid

Lastly, one remaining study, Diel studied the value of delamanid and found that addition of the drug to existing background regimens dominated existing background regimens in a German setting with an ICER of 3,494 EUR/QALY at a threshold of 10,000 EUR (24). However, as Diel was the only study in our sample to study Delamanid, it is difficult to draw conclusions on the cost-effectiveness of delamanid or its future role in treatment regimens (24).

### Sensitivity analysis

Probabilistic and deterministic sensitivity analyses found that the cost-effectiveness determinations were largely stable, but were sensitive to several key factors depending on the intervention being evaluated. For adding bedaquiline to background or standard regimens, cost-effectiveness determinations were most sensitive to the number of TB cases and the proportion of cases with sputum culture conversion (25, 27). For studies evaluating the cost-effectiveness of shorter versus longer regimens for MDR-TB, results were most-sensitive to existing health service costs, drug costs, treatment delivery costs, and treatment efficacy in limiting recurrent-TB mortality (37, 38).

### Findings from HTA reports

Lastly, the HTA reports found similar results to the aforementioned studies. The HTA report by EUNEHTA in 2020 found that the BpaL regimen had a high rate of cost-effectiveness relative to existing background regimens (42). The report from the German G-BA comparing bedaquiline to an unspecified background regimen found a non-quantifiable additional benefit from the addition of bedaquiline (41). The HTA report by the All Wales Medicines Strategy Group (AWMSG) similarly recommended bedaquiline over multiple background regimens (40).

## Discussion

This study synthesizes 20 economic evaluations and 3 HTA reports evaluating various pharmacological interventions for TB. Heterogeneity and variation in studies prevent the quantitative synthesis of our results, but several broad trends are observable.

For the addition of bedaquiline to background regimens or standard regimens for TB, clinical evidence on bedaquiline’s efficacy has been limited (43). However, nearly all studies in our review found the addition of bedaquiline to existing background or standard regimens against drug-resistant TB was cost-effective across a variety of international settings (22, 25, 27, 29–34, 38). Bedaquiline also became cost-effective in a greater number of cases if analyses accounted for adverse reactions (30). Bedaquiline’s high cost-effectiveness, thus, despite limited clinical evidence, may justify a slightly higher value-based price. Similarly, this result highlights a need for effective antimicrobial stewardship to limit the prevalence of bedaquiline-resistant strains and maintain the drug’s effectiveness and, hence, its cost-effectiveness.

For the question of short-course versus long-course treatment regimens, our findings broadly support the conclusion that shorter-course regimens may be more effective, even if a shorter-course regimen was more expensive than its longer counterpart. Even in Reddy et al., where a longer regimen dominated a shorter one, suggested that a shorter-regimen may be more cost-effective if patient loss to follow-up/drop out was accounted for in the model. (44) This result suggests that evidence-based clinical guidelines could consider shortening treatment regimens, especially in low-resource settings, to maximize the number of lives saved given limited resources. Lastly, our findings for ethambutol finds that addition of the drug to existing TB regimens is not likely to be cost-effective, but our analysis is based on solely two studies and hence are not conclusive of the drug’s value (19, 20).

This review also highlights more specific gaps in current economic evaluations of treatments for active tuberculosis. First, most studies did not consider the transmission costs of TB in their models — a uniquely problematic oversight due to the disease’s high infectivity and latency periods (1–3). Acc-ounting for these transmission costs may alter the cost-effectiveness of various TB treatments depending on the stage at which interventions successfully treat TB. Second, no study accounted for the potential costs of AMR— an important and common oversight for antimicrobial drugs like bedaquiline, where the treatment’s relative novelty should create an impetus for proper stewardship policies to limit the advent of drug-resistant strains. Third, only one study, Schippel et al. accounted for the costs of adverse reactions (30). Given that many standard tuberculosis regimens can have several adverse effects, and even new drugs like bedaquiline can induce corrected QT prolongations, hyperlactatemia, and more, accounting for such effects is integral to future cost-effectiveness analyses (45).

Beyond specific interventions for TB, this systematic review highlights a strong need for novel pharmacological interventions for active TB. Of the interventions in our review, the only drug with favorable cost-effectiveness across multiple settings was bedaquiline, with other interventions displaying more unclear results. Given that bedaquiline is a single drug against which resistant strains have already emerged, our results highlight a need for pharmaceutical companies and governments to accelerate the development of novel MDR-TB and XDR-TB treatments (46). Such developments would help ease reliance on bedaquiline as a last-resort treatment for drug-resistant TB and may prove more efficacious in treating future drug-resistant strains.

The HTA reports find similar results to those in the studies. Two out of three HTA reports deemed bedaquiline to be cost-effective compared to existing comparators and background regimens (40–42). The third report similarly found bedaquiline to be cost-effective as part of a combination BPaL regimen, but further studies are likely needed to more strongly characterize BpaL’s cost-effectiveness relative to existing background regimens (40–42). Additionally worth noting is that many core elements of value — including scientific spillovers, productivity effects, family spillovers, equity, and more were not discussed by any HTA reports. This result may highlight a current deficiency with existing HTA appraisal strategies, and suggests that existing HTA agencies should consider considering additional elements of value in appraisal decisions. However, given that our sample size is limited, we cannot make a definitive conclusion on this matter.

### Limitations

This review has a number of limitations. We excluded studies that were not published in English, were abstracts with insufficient detail or were preprints. Our decision to omit such studies may have limited our ability to identify papers from foreign nations, especially in several non-English countries which may have TB incidence, influencing the results of our analysis (8). Lastly, our analysis is certainly not definitive as to the cost-effectiveness of various pharmacological interventions for TB. There is limited clinical evidence for the efficacy of drugs like bedaquiline, and as new pharmacological interventions are developed, and new data emerges, such findings are likely to influence cost-effectiveness determinations. Moreover, conducting economic evaluations in the context of anti-tuberculosis interventions can often face difficulties, such as limited data on the efficacy of a given intervention vs. a comparator (23).

Despite these limitations, this review provides the most comprehensive overview of the economic evidence available relating to cost effectiveness of pharmacological treatments of active TB, the key drivers of the results and the additional value attributes to consider when assessing these treatments by HTA agencies. Optimizing the development of novel treatment regimens to address these value attributes will be key in ensuring positive assessment outcomes, which in turn will lead to prompt patient access to these treatments particularly in resource limited settings.

## Conclusion

Our review of economic evaluations of pharmacological interventions for active drug-resistant TB shows that the addition of bedaquiline to existing background or standard regimens for drug-resistant TB is likely to be highly cost-effective across a number of international settings. However, such findings are tempered by the limited clinical evidence collected on the real-world effectiveness of bedaquiline. It also shows that shortening TB treatment regimens, especially in low-resource settings, may be a cost-effective strategy, while we lack sufficient evidence to draw strong conclusions about the cost-efficacy of other drugs like ethambutol and delamanid.

These results highlight a need for both the private and public sector to support the development of novel antimicrobial treatments against active drug-resistant TB, especially given the limited number of cost-effective treatments for MDR-TB and XDR-TB present at this time. Our results also highlight a growing need for economic modeling to consider the costs of transmission, antimicrobial resistance, and adverse events, especially given the relevance of all three cost categories for pharmacological interventions against active TB. Lastly, our results also suggest that in certain resource poor settings, scientists ought to consider the feasibility of shortening treatment regimens to maximize the number of lives saved while ensuring efficient allocation of resources. Such policy steps could ensure that the world has the necessary innovation and resources to combat drug-resistant tuberculosis in an evidence-based and equitable manner.

## Data availability statement

The original contributions presented in the study are included in the article/Supplementary material, further inquiries can be directed to the corresponding author.

## Author contributions

SN: Data curation, Methodology, Conceptualization, Formal analysis, Writing – original draft. DN: Data curation, Methodology, Writing – review & editing. DD: Data curation, Methodology, Supervision, Conceptualization, Formal analysis, Validation, Funding acquisition, Resources, Writing – review & editing.
