# Supplementary material for: A systematic review of economic evaluations of pharmacological treatments for active tuberculosis
Source: Front Public Health. 2024 Apr 16;12:1201512. doi: 10.3389/fpubh.2024.1201512 (PMC11060080; doi:10.3389/fpubh.2024.1201512)
Supplement: Supplementary file 1 [file Data_Sheet_1.docx]

**Study 1:** Codecasa et al. 2017

| **Section 2: Study limitations** (the level of methodological quality)  This checklist should be used once it has been decided that the study is sufficiently applicable to the context of the guideline | **Yes/partly/no/unclear/NA** | **Comments** |
| --- | --- | --- |
| 2.1 Does the model structure adequately reflect the nature of the topic under evaluation? | Yes | Yes; logically makes sense |
| 2.2 Is the time horizon sufficiently long to reflect all important differences in costs and outcomes? | Partly | The model is over a 10-year, rather than lifelong, |
| 2.3 Are all important and relevant outcomes included? | Partly | Did not include transmission and impact on resistance as well as adverse effects of injectable drugs |
| 2.4 Are the estimates of baseline outcomes from the best available source? | No | placebo arm of the trials |
| 2.5 Are the estimates of relative intervention effects from the best available source? | No | Data from the treatment effects in the XDR-TB cohort were based on the C209 study, which was an open-label, single-arm study. |
| 2.6 Are all important and relevant costs included? | Yes | Costs of hospitalization, drug regimen, end of life costs, and changes in productivity are included |
| 2.7 Are the estimates of resource use from the best available source? | Yes | From Italian NHS and other sources. |
| 2.8 Are the unit costs of resources from the best available source? | Partly | For some drugs, the entire NHS prices are used, but for other drugs, prices from only two centers are used. |
| 2.9 Is an appropriate incremental analysis presented or can it be calculated from the data? | Yes | ICERs are calculated. |
| 2.10 Are all important parameters whose values are uncertain subjected to appropriate sensitivity analysis? | Yes | Both deterministic and probabilistic. |
| 2.11 Has no potential financial conflict of interest been declared? | No | Several study authors are funded by Janssen . |
| 2.12 **Overall assessment:** Minor limitations/potentially serious limitations/very serious limitations.  Study was deemed to have potentially serious limitations because not all unit costs were included, not all outcomes were included, and because baseline estimates and relative intervention effects were not estimated from gold-standard studies. | | |
| Other comments | | |

**Study 2: Lu et al 2017**

| **Section 2: Study limitations** (the level of methodological quality)  This checklist should be used once it has been decided that the study is sufficiently applicable to the context of the guideline | **Yes/partly/no/unclear/NA** | **Comments** |
| --- | --- | --- |
| 2.1 Does the model structure adequately reflect the nature of the topic under evaluation? | Yes |  |
| 2.2 Is the time horizon sufficiently long to reflect all important differences in costs and outcomes? | Partly | Model uses only a 10-year time horizon, however mortality was not measured. a lifetime horizon is still the preferred approach especially if they would have used the results of the trial that showed that mortality was higher in the bedaquiline arm |
| 2.3 Are all important and relevant outcomes included? | Partly | Did not include transmission and impact on resistance as well as adverse effects of injectable drugs |
| 2.4 Are the estimates of baseline outcomes from the best available source? | Partly | Data comes from a single-RCT of MDR-TB patients, with some country data also used. |
| 2.5 Are the estimates of relative intervention effects from the best available source? | Partly | Data comes from a single-RCT of MDR-TB patients, with some country data also used. Some data also came from the open-label C209 study. |
| 2.6 Are all important and relevant costs included? | Partly | Testing costs are not considered. Also there is not a specific breakdown of BR costs, so this is somewhat unclear. End-of-life care costs do not seem to be included in the table, nor are transmission costs. Treatment acquisition costs were not considered because such data was not available. |
| 2.7 Are the estimates of resource use from the best available source? | Yes | Resource use costs came from official government sources with supplementation from other credible sources, like systematic reviews. |
| 2.8 Are the unit costs of resources from the best available source? | Yes | Yes, unit costs came from government sources with supplementation from other credible sources, like systematic reviews. |
| 2.9 Is an appropriate incremental analysis presented or can it be calculated from the data? | Yes | ICERs are presented. |
| 2.10 Are all important parameters whose values are uncertain subjected to appropriate sensitivity analysis? | Yes | A probabilistic sensitivity analysis was performed |
| 2.11 Has no potential financial conflict of interest been declared? | No | The study was funded by JANSSEN. |
| 2.12 **Overall assessment:** Minor limitations/potentially serious limitations/very serious limitations  This study had potentially serious limitations due to a poor source of relative intervention effects and outcomes, a limited time horizon, and not all costs were considered. | | |
| Other comments | | |

**Study 3: Park et al 2016**

| **Section 2: Study limitations** (the level of methodological quality)  This checklist should be used once it has been decided that the study is sufficiently applicable to the context of the guideline | **Yes/partly/no/unclear/NA** | **Comments** |
| --- | --- | --- |
| 2.1 Does the model structure adequately reflect the nature of the topic under evaluation? | Yes |  |
| 2.2 Is the time horizon sufficiently long to reflect all important differences in costs and outcomes? | Partly | 20-year time horizon |
| 2.3 Are all important and relevant outcomes included? | Partly | Did not include transmission and impact on resistance as well as adverse effects of injectable drugs |
| 2.4 Are the estimates of baseline outcomes from the best available source? | Partly | All outcomes came from a single RCT, the C208 study |
| 2.5 Are the estimates of relative intervention effects from the best available source? | Partly | All outcomes came from a single RCT, the C208 study |
| 2.6 Are all important and relevant costs included? | Yes | Both indirect and direct costs, including for monitoring, end of life care, productivity, and more were included. |
| 2.7 Are the estimates of resource use from the best available source? | Yes | The resource costs and the unit costs were obtained from the Korean government’s HIRA guidelines. |
| 2.8 Are the unit costs of resources from the best available source? | Yes | The resource costs and the unit costs were obtained from the Korean government’s HIRA guidelines. |
| 2.9 Is an appropriate incremental analysis presented or can it be calculated from the data? | Yes | ICERs were calculated |
| 2.10 Are all important parameters whose values are uncertain subjected to appropriate sensitivity analysis? | Yes | Probabilistic sensitivity analyses were conducted |
| 2.11 Has no potential financial conflict of interest been declared? | No | JANSSEN funded the study. |
| 2.12 **Overall assessment:** Minor limitations/potentially serious limitations/very serious limitations.  This study was found to have potentially serious limitations due to the fact that the time horizon was limited, not all outcomes were included, and the source of relative intervention effects was not available. | | |
| Other comments | | |

**Study 4: Schnippel a**

| **Section 2: Study limitations** (the level of methodological quality)  This checklist should be used once it has been decided that the study is sufficiently applicable to the context of the guideline | **Yes/partly/no/unclear/NA** | **Comments** |
| --- | --- | --- |
| 2.1 Does the model structure adequately reflect the nature of the topic under evaluation? | Yes | Model is logical and rooted in data |
| 2.2 Is the time horizon sufficiently long to reflect all important differences in costs and outcomes? | Partly | 10-year time horizon |
| 2.3 Are all important and relevant outcomes included? | partly | Did not include transmission and impact on resistance as well as adverse effects of injectable drugs |
| 2.4 Are the estimates of baseline outcomes from the best available source? | Yes | The baseline outcomes are from a broad observational study of patients in in the South African National TB Program (NTP) |
| 2.5 Are the estimates of relative intervention effects from the best available source? | No | The clinical outcomes are not based on RCTs and come from observational data of patients in SA NTP |
| 2.6 Are all important and relevant costs included? | Yes | Testing costs, drug costs, and other investigative costs are accounted for. |
| 2.7 Are the estimates of resource use from the best available source? | Yes | National level sources were used |
| 2.8 Are the unit costs of resources from the best available source? | Yes | National level sources were used |
| 2.9 Is an appropriate incremental analysis presented or can it be calculated from the data? | Yes | ICERs are presented. |
| 2.10 Are all important parameters whose values are uncertain subjected to appropriate sensitivity analysis? | Partly | Only deterministic analysis is conducted. |
| 2.11 Has no potential financial conflict of interest been declared? | Yes |  |
| 2.12 **Overall assessment:** Minor limitations/potentially serious limitations/very serious limitations  This study had potentially serious limitations because relative intervention effects did not come from the best source, all important and relevant outcomes were not included, and the time horizon was not sufficiently long. | | |
| Other comments | | |

**Study 5: Schnippel b**

| **Section 2: Study limitations** (the level of methodological quality)  This checklist should be used once it has been decided that the study is sufficiently applicable to the context of the guideline | **Yes/partly/no/unclear/NA** | **Comments** |
| --- | --- | --- |
| 2.1 Does the model structure adequately reflect the nature of the topic under evaluation? | Yes |  |
| 2.2 Is the time horizon sufficiently long to reflect all important differences in costs and outcomes? | Partly | Time horizon of 10 years despite dealing with mortality |
| 2.3 Are all important and relevant outcomes included? | Partly | transmission and resistance not included |
| 2.4 Are the estimates of baseline outcomes from the best available source? | Yes | Yes — baseline outcomes from a broad, national-level registry in South Africa |
| 2.5 Are the estimates of relative intervention effects from the best available source? | No | There are issues with the estimates — two of the drugs seem to be different in RCT, even though they were assumed to be the same, and the data for the rest of the interventions was from an observational study. |
| 2.6 Are all important and relevant costs included? | Yes | Yes — all major costs, including testing, treatment, and more |
| 2.7 Are the estimates of resource use from the best available source? | Yes | Resource use costs are found from the South African government |
| 2.8 Are the unit costs of resources from the best available source? | Yes | Unit costs are from the South African government |
| 2.9 Is an appropriate incremental analysis presented or can it be calculated from the data? | Yes | Incremental cost calculated |
| 2.10 Are all important parameters whose values are uncertain subjected to appropriate sensitivity analysis? | Unclear | Only one variable is explicitly mentioned as being analyzed, however, it is implied there are further analyses and the type of analysis is unclear |
| 2.11 Has no potential financial conflict of interest been declared? | Yes |  |
| 2.12 **Overall assessment:** Minor limitations/potentially serious limitations/very serious limitations  This study had potentially serious limitations due to the fact that the estimates of relative intervention effects are not available, important outcomes were not included, and time horizon was not long enough. | | |
| Other comments | | |

**Study 6: Fan et al. 2019**

| **Section 2: Study limitations** (the level of methodological quality)  This checklist should be used once it has been decided that the study is sufficiently applicable to the context of the guideline | **Yes/partly/no/unclear/NA** | **Comments** |
| --- | --- | --- |
| 2.1 Does the model structure adequately reflect the nature of the topic under evaluation? | Yes |  |
| 2.2 Is the time horizon sufficiently long to reflect all important differences in costs and outcomes? | Partly | Time-horizon is only 10 years long, but the study deals with mortality |
| 2.3 Are all important and relevant outcomes included? | Yes |  |
| 2.4 Are the estimates of baseline outcomes from the best available source? | Yes | Base case outcomes came from the government of Hong Kong. |
| 2.5 Are the estimates of relative intervention effects from the best available source? | Yes | Interventions come from a meta-analysis of 351 patients, a randomized control trial, and an observational study with two arms for three of the drugs. Two out of three of these are rigorous, and the observational study was very large (25k+) patients, so quality is high. |
| 2.6 Are all important and relevant costs included? | Partly | Costs of testing not included, but other costs are kept. |
| 2.7 Are the estimates of resource use from the best available source? | Yes | Resource use costs came from a study, this is acceptable. |
| 2.8 Are the unit costs of resources from the best available source? | Yes | Unit costs came from “local cost” — but because this Hong Kong, local cost is likely sufficient |
| 2.9 Is an appropriate incremental analysis presented or can it be calculated from the data? | Yes | Incremental cost analyses were calculated |
| 2.10 Are all important parameters whose values are uncertain subjected to appropriate sensitivity analysis? | Yes | Probabilistic sensitivity analysis was performed |
| 2.11 Has no potential financial conflict of interest been declared? | Yes |  |
| 2.12 **Overall assessment:** Minor limitations/potentially serious limitations/very serious limitations | | |
| Other comments | | |

**Study 7:** Wirth et al. 2017

| **Section 2: Study limitations** (the level of methodological quality)  This checklist should be used once it has been decided that the study is sufficiently applicable to the context of the guideline | **Yes/partly/no/unclear/NA** | **Comments** |
| --- | --- | --- |
| 2.1 Does the model structure adequately reflect the nature of the topic under evaluation? | Yes |  |
| 2.2 Is the time horizon sufficiently long to reflect all important differences in costs and outcomes? | Partly | Model uses a 10-year time horizon despite dealing with mortality |
| 2.3 Are all important and relevant outcomes included? | Yes | All major outcomes — death, follow-up, sputum, etc are seen |
| 2.4 Are the estimates of baseline outcomes from the best available source? | Partly | Several studies are cited, but all are individual studies, and there are assumptions made with mortality. |
| 2.5 Are the estimates of relative intervention effects from the best available source? | Partly | Efficacy of some interventions from observational studies, other from trials, so partly. |
| 2.6 Are all important and relevant costs included? | Yes | Acquisition costs, testing costs, and other resource costs appear to be included in the model |
| 2.7 Are the estimates of resource use from the best available source? | Partly | Resource use was not from a government source, but an independent analysis of pan-German data. |
| 2.8 Are the unit costs of resources from the best available source? | Yes | Unit costs were sourced from the German government. |
| 2.9 Is an appropriate incremental analysis presented or can it be calculated from the data? | Yes | Incremental costs were calculated |
| 2.10 Are all important parameters whose values are uncertain subjected to appropriate sensitivity analysis? | Yes | Both deterministic and probabilistic sensitivity analyses were conducted. |
| 2.11 Has no potential financial conflict of interest been declared? | No | Study commissioned by JANSSEN |
| 2.12 **Overall assessment:** Minor limitations/potentially serious limitations/very serious limitations  This study had potentially serious limitations due to the fact that its relative intervention effects did not come from the best possible source, resource use costs were not from the best source, and time horizons were limited. | | |
| Other comments | | |

**Study 8: Wolfson et al. 2015**

| **Section 2: Study limitations** (the level of methodological quality)  This checklist should be used once it has been decided that the study is sufficiently applicable to the context of the guideline | **Yes/partly/no/unclear/NA** | **Comments** |
| --- | --- | --- |
| 2.1 Does the model structure adequately reflect the nature of the topic under evaluation? | Yes |  |
| 2.2 Is the time horizon sufficiently long to reflect all important differences in costs and outcomes? | Partly | The time horizon is 10 years, despite dealing with mortality |
| 2.3 Are all important and relevant outcomes included? | Yes | All outcomes – death, conversion, surgery, reversion, and death are accounted for. |
| 2.4 Are the estimates of baseline outcomes from the best available source? | Partly | All outcomes came from a single RCT, the C208 study |
| 2.5 Are the estimates of relative intervention effects from the best available source? | Partly | All outcomes came from a single RCT, the C208 study |
| 2.6 Are all important and relevant costs included? | Yes | Major costs of treatment, course, monitoring, hospitalization, surgery and more are found. |
| 2.7 Are the estimates of resource use from the best available source? | Partly | Resource use costs were based on a co-authors’ opinion and an interview, rather than a study |
| 2.8 Are the unit costs of resources from the best available source? | Yes | Unit costs were sourced from the UK national drug formulary. |
| 2.9 Is an appropriate incremental analysis presented or can it be calculated from the data? | Yes | Incremental costs were calculated |
| 2.10 Are all important parameters whose values are uncertain subjected to appropriate sensitivity analysis? | Yes | Probabilistic sensitivity analysis was conducted |
| 2.11 Has no potential financial conflict of interest been declared? | No | This study was sponsored by JANSSEN |
| 2.12 **Overall assessment:** Minor limitations/potentially serious limitations/very serious  This study had potentially serious limitations due to the fact that the estimates for resource use, relative intervention effects, and baseline outcomes did not come from the best possible source. | | |
| Other comments | | |

**Study 9: Gomez et al. 2016**

| **Section 2: Study limitations** (the level of methodological quality)  This checklist should be used once it has been decided that the study is sufficiently applicable to the context of the guideline | **Yes/partly/no/unclear/NA** | **Comments** |
| --- | --- | --- |
| 2.1 Does the model structure adequately reflect the nature of the topic under evaluation? | Yes |  |
| 2.2 Is the time horizon sufficiently long to reflect all important differences in costs and outcomes? | No | The model takes place over several months, despite the fact it deals with mortality |
| 2.3 Are all important and relevant outcomes included? | Yes | All major states (mortality, disability, etc) was found and analyzed. |
| 2.4 Are the estimates of baseline outcomes from the best available source? | Partly | Several values are assumed. But many clinical probabilities appear to be based on systematic review data, or published research. |
| 2.5 Are the estimates of relative intervention effects from the best available source? | No | based on an assumption on non-inferiority |
| 2.6 Are all important and relevant costs included? | Yes | All major costs, including first-time treatment and testing costs are included |
| 2.7 Are the estimates of resource use from the best available source? | Yes | Resource use estimates come from different studies across various countries |
| 2.8 Are the unit costs of resources from the best available source? | Partly | Unit costs come from trials rather than government sources |
| 2.9 Is an appropriate incremental analysis presented or can it be calculated from the data? | Yes | Incremental costs were calculated |
| 2.10 Are all important parameters whose values are uncertain subjected to appropriate sensitivity analysis? | Yes | Probabilistic sensitivity analyses |
| 2.11 Has no potential financial conflict of interest been declared? | Yes | Authors affiliated with nonprofit organizations during study |
| 2.12 **Overall assessment:** Minor limitations/potentially serious limitations/very serious limitations  This study had very serious limitations due to a bad time horizon, poor sources for estimates of baseline outcomes, and poor sources for relative intervention effects and unit costs. | | |
| Other comments | | |

**Study 10: Knight et al. 2015**

| **Section 2: Study limitations** (the level of methodological quality)  This checklist should be used once it has been decided that the study is sufficiently applicable to the context of the guideline | **Yes/partly/no/unclear/NA** | **Comments** |
| --- | --- | --- |
| 2.1 Does the model structure adequately reflect the nature of the topic under evaluation? | Yes |  |
| 2.2 Is the time horizon sufficiently long to reflect all important differences in costs and outcomes? | Partly | The time horizon is 20 years, but study includes mortality as an outcome |
| 2.3 Are all important and relevant outcomes included? | yes | All major outcomes are accounted for (disease, death, disability, etc). |
| 2.4 Are the estimates of baseline outcomes from the best available source? | Yes | Baseline outcomes from previous studies and the WHO. |
| 2.5 Are the estimates of relative intervention effects from the best available source? | No | Assumption of non-inferiority |
| 2.6 Are all important and relevant costs included? | Yes | All major costs, including the cost of resistance developing, is accounted for |
| 2.7 Are the estimates of resource use from the best available source? | Yes | Costs from costing surveys in previous studies |
| 2.8 Are the unit costs of resources from the best available source? | Partly | Unit costs came from a study across South Africa, but not from the government |
| 2.9 Is an appropriate incremental analysis presented or can it be calculated from the data? | Partly/Unclear | An incremental value was calculated as a willingness to pay, but it is unclear where this is in the paper. The incremental value was also calculated for costs, not cost effectiveness, and it is unclear if it can be derived from the data. |
| 2.10 Are all important parameters whose values are uncertain subjected to appropriate sensitivity analysis? | Partly | They did a form of scenario analysis, saying it would have larger results than a PSA (which would have been more computationally intensive)/ This is a form of deterministic model. |
| 2.11 Has no potential financial conflict of interest been declared? | Yes |  |
| 2.12 **Overall assessment:** Minor limitations/potentially serious limitations/very serious limitations  This study has potentially serious limitations as it has major clarity problems when being read, as well as that its relative effectiveness is based on an assumption. | | |
| Other comments | | |

**Study 11: Law et al 2014**

| **Section 2: Study limitations** (the level of methodological quality)  This checklist should be used once it has been decided that the study is sufficiently applicable to the context of the guideline | **Yes/partly/no/unclear/NA** | **Comments** |
| --- | --- | --- |
| 2.1 Does the model structure adequately reflect the nature of the topic under evaluation? | Yes |  |
| 2.2 Is the time horizon sufficiently long to reflect all important differences in costs and outcomes? | Partly | 10 year analytical horizon used despite dealing with mortality |
| 2.3 Are all important and relevant outcomes included? | Yes |  |
| 2.4 Are the estimates of baseline outcomes from the best available source? | Partly | Baseline outcomes came from the WHO and other organizations — but assumptions were made across types of diseases |
| 2.5 Are the estimates of relative intervention effects from the best available source? | Partly | Relative intervention outcomes came from an WHO review of datam but an assumption about MDR-TB and intervention effectiveness was made |
| 2.6 Are all important and relevant costs included? | Yes | Societal perspective |
| 2.7 Are the estimates of resource use from the best available source? | Yes | Resource use levels were primarily based on a cost survey, but this had a large enough sample size |
| 2.8 Are the unit costs of resources from the best available source? | Partly | It is from the government of Ecuador, but they said the study was to be used more generally, which may not be possible given this data. |
| 2.9 Is an appropriate incremental analysis presented or can it be calculated from the data? | Yes | ICERs were presented |
| 2.10 Are all important parameters whose values are uncertain subjected to appropriate sensitivity analysis? | Yes | A full probabilistic sensitivity analysis was conducted |
| 2.11 Has no potential financial conflict of interest been declared? | Yes |  |
| 2.12 **Overall assessment:** Minor limitations/potentially serious limitations/very serious limitations | | |
| Other comments | | |

**Study 12: Manabe et al 2012**

| **Section 2: Study limitations** (the level of methodological quality)  This checklist should be used once it has been decided that the study is sufficiently applicable to the context of the guideline | **Yes/partly/no/unclear/NA** | **Comments** |
| --- | --- | --- |
| 2.1 Does the model structure adequately reflect the nature of the topic under evaluation? | Yes |  |
| 2.2 Is the time horizon sufficiently long to reflect all important differences in costs and outcomes? | No/Unclear | The time horizon is difficult to find or not specified, but it appears to be at the end of the treatment regimen |
| 2.3 Are all important and relevant outcomes included? | Yes | All major clinical endpoints are represented |
| 2.4 Are the estimates of baseline outcomes from the best available source? | Yes | Based on observational studies |
| 2.5 Are the estimates of relative intervention effects from the best available source? | Yes | Intervention effects are from a systematic review and meta-analysis |
| 2.6 Are all important and relevant costs included? | Partly | Costs of diagnostics, hospitalization, and more do not seem to be included |
| 2.7 Are the estimates of resource use from the best available source? | Partly | Costs were estimated from surveys and mathematically computed from a single survey site. |
| 2.8 Are the unit costs of resources from the best available source? | Partly | Unit costs seem derived from studies in other countries and rely on assumptions. |
| 2.9 Is an appropriate incremental analysis presented or can it be calculated from the data? | No | No incremental cost-effectiveness work was calculated |
| 2.10 Are all important parameters whose values are uncertain subjected to appropriate sensitivity analysis? | Partly | A deterministic sensitivity analysis was performed |
| 2.11 Has no potential financial conflict of interest been declared? | Yes |  |
| 2.12 **Overall assessment:** Minor limitations/potentially serious limitations/very serious limitations  This study has potentially serious limitations due to its unclear time horizons, poor sources of resource use and unit costs, and lack of inclusion of relevant costs. | | |
| Other comments | | |

**Study 13: Owens et al 2013**

| **Section 2: Study limitations** (the level of methodological quality)  This checklist should be used once it has been decided that the study is sufficiently applicable to the context of the guideline | **Yes/partly/no/unclear/NA** | **Comments** |
| --- | --- | --- |
| 2.1 Does the model structure adequately reflect the nature of the topic under evaluation? | Yes |  |
| 2.2 Is the time horizon sufficiently long to reflect all important differences in costs and outcomes? | Yes | Lifetime time horizon with mortality used |
| 2.3 Are all important and relevant outcomes included? | Yes | Decision tree model is accurately captures the main outcomes of treatment |
| 2.4 Are the estimates of baseline outcomes from the best available source? | Yes | Data based on WHO information about treatment regimens, and assumptions were tested with sensitivity analyses. |
| 2.5 Are the estimates of relative intervention effects from the best available source? | Partly | Based on assumption of equivalence |
| 2.6 Are all important and relevant costs included? | Partly | Treatment costs are mainly included |
| 2.7 Are the estimates of resource use from the best available source? | Partly | Resource costs are effectively calculated, but many assumptions are made, but they make multiple scenarios to test different assumptions |
| 2.8 Are the unit costs of resources from the best available source? | Yes | Unit costs from the WHO which makes it globally generalizable |
| 2.9 Is an appropriate incremental analysis presented or can it be calculated from the data? | Yes | Incremental costs were calculated |
| 2.10 Are all important parameters whose values are uncertain subjected to appropriate sensitivity analysis? | Partly | It appears a multiway analysis was done, but the graph is a Tornado diagram and the analysis is deterministic |
| 2.11 Has no potential financial conflict of interest been declared? | Yes | Funding from NIA, Global Alliance for TB Research provided funding, but only after research was concluded. |
| 2.12 **Overall assessment:** Minor limitations/potentially serious limitations/very serious limitations  This study has potentially serious limitations due to sources of resource use not being for the best available source, relative intervention effects not being from the best available source, and other important costs not being included. | | |
| Other comments | | |

**Study 14: Manalan et al, 2020**

| **Section 2: Study limitations** (the level of methodological quality)  This checklist should be used once it has been decided that the study is sufficiently applicable to the context of the guideline | **Yes/partly/no/unclear/NA** | **Comments** |
| --- | --- | --- |
| 2.1 Does the model structure adequately reflect the nature of the topic under evaluation? | NA | Cost-comparison study |
| 2.2 Is the time horizon sufficiently long to reflect all important differences in costs and outcomes? | No | short retrospective follow up |
| 2.3 Are all important and relevant outcomes included? | Partly | assumed equivalent |
| 2.4 Are the estimates of baseline outcomes from the best available source? | Yes | Data were collected directly from cohort of patients in a retrospective observational study |
| 2.5 Are the estimates of relative intervention effects from the best available source? | Partly | assumed equivalent |
| 2.6 Are all important and relevant costs included? | No | Many relevant testing and staff costs are not accounted for |
| 2.7 Are the estimates of resource use from the best available source? | Yes | Cohort of patients observed in the sample |
| 2.8 Are the unit costs of resources from the best available source? | Partly | Drug Tariff some, both others sourced from hospitals |
| 2.9 Is an appropriate incremental analysis presented or can it be calculated from the data? | Yes | No incremental costs were calculated, but subtraction can find differences between interventions |
| 2.10 Are all important parameters whose values are uncertain subjected to appropriate sensitivity analysis? | Partly | Some scenario changes are modeled, but other key ones are missing (i.e. if bedaquiline pricing falls). |
| 2.11 Has no potential financial conflict of interest been declared? | No |  |
| 2.12 **Overall assessment:** Minor limitations/potentially serious limitations/very serious limitations  This study has potentially serious limitations due to the fact that relative intervention effects did not come from the best available source, and all important and relevant costs were not included. | | |
| Other comments | | |

**Study 15: Gomez et al, 2021.**

| **Section 2: Study limitations** (the level of methodological quality)  This checklist should be used once it has been decided that the study is sufficiently applicable to the context of the guideline | **Yes/partly/no/unclear/NA** | **Comments** |
| --- | --- | --- |
| 2.1 Does the model structure adequately reflect the nature of the topic under evaluation? | Yes | Markov model |
| 2.2 Is the time horizon sufficiently long to reflect all important differences in costs and outcomes? | Yes | Lifetime horizon |
| 2.3 Are all important and relevant outcomes included? | Yes | All relevant outcomes are included |
| 2.4 Are the estimates of baseline outcomes from the best available source? | Partly | Baseline outcomes are based on national secondary data and Expert elicitation was used where no data were available |
| 2.5 Are the estimates of relative intervention effects from the best available source? | No | open-label single-arm study |
| 2.6 Are all important and relevant costs included? | Partly | Testing costs and other key costs are not considered. |
| 2.7 Are the estimates of resource use from the best available source? | Partly | Resource use estimates come from the literature, surveys, and studies. |
| 2.8 Are the unit costs of resources from the best available source? | Partly | Unit costs do not come from governments, but come from surveys across countries. |
| 2.9 Is an appropriate incremental analysis presented or can it be calculated from the data? | Yes | ICER calculated |
| 2.10 Are all important parameters whose values are uncertain subjected to appropriate sensitivity analysis? | Yes | PSA was performed |
| 2.11 Has no potential financial conflict of interest been declared? | Yes | Study was funded by Bill & Melinda Gates foundation and TB Alliance |
| 2.12 **Overall assessment:** Minor limitations/potentially serious limitations/very serious limitations  This study had potentially serious limitations due to the fact that relative intervention effects, resource use estimates, and unit costs did not come from the best source. | | |
| Other comments | | |

**Study 16: Agnarson et al, 2020.**

| **Section 2: Study limitations** (the level of methodological quality)  This checklist should be used once it has been decided that the study is sufficiently applicable to the context of the guideline | **Yes/partly/no/unclear/NA** | **Comments** |
| --- | --- | --- |
| 2.1 Does the model structure adequately reflect the nature of the topic under evaluation? | Yes |  |
| 2.2 Is the time horizon sufficiently long to reflect all important differences in costs and outcomes? | No | 10-year time horizon |
| 2.3 Are all important and relevant outcomes included? | Yes | Mortality, disability and other key states are all accounted for. |
| 2.4 Are the estimates of baseline outcomes from the best available source? | Yes | Baseline outcomes from multiple natural history studies |
| 2.5 Are the estimates of relative intervention effects from the best available source? | Partly | Intervention effects are pulled from a wide variety of studies, but many are individual studies from different contexts, and there was not a systematic review for intervention effects. |
| 2.6 Are all important and relevant costs included? | Yes | foremost relevant categories captured |
| 2.7 Are the estimates of resource use from the best available source? | Yes | Resource use for patients came from South African government, WHO, and other studies |
| 2.8 Are the unit costs of resources from the best available source? | Yes | Unit costs are from national governments & official medicinal pricing information from IGOs |
| 2.9 Is an appropriate incremental analysis presented or can it be calculated from the data? | Yes | Yes, an incremental analysis was conducted |
| 2.10 Are all important parameters whose values are uncertain subjected to appropriate sensitivity analysis? | Yes | PSA was performed |
| 2.11 Has no potential financial conflict of interest been declared? | No | Study by Johnson & Johnson |
| 2.12 **Overall assessment:** Minor limitations/potentially serious limitations/very serious limitations  This study has potentially serious limitations due to the fact that relative intervention effects did not come from the best source, due to poor time horizons, and potential financial conflicts of interest. | | |
| Other comments | | |

**Study 17: Madan et al, 2020.**

| **Section 2: Study limitations** (the level of methodological quality)  This checklist should be used once it has been decided that the study is sufficiently applicable to the context of the guideline | **Yes/partly/no/unclear/NA** | **Comments** |
| --- | --- | --- |
| 2.1 Does the model structure adequately reflect the nature of the topic under evaluation? | Not applicable | No model. Data came from RCT, from which cost-effectiveness was estimated. |
| 2.2 Is the time horizon sufficiently long to reflect all important differences in costs and outcomes? | No | Time horizon was 132 weeks |
| 2.3 Are all important and relevant outcomes included? | Unclear | no outcome data reported from the trial |
| 2.4 Are the estimates of baseline outcomes from the best available source? | Unclear | no outcome data reported from the trial |
| 2.5 Are the estimates of relative intervention effects from the best available source? | Unclear | no outcome data reported from the trial |
| 2.6 Are all important and relevant costs included? | Yes | All relevant costs, even beyond those provided in the clinical trial, were collected. |
| 2.7 Are the estimates of resource use from the best available source? | Yes | Resource use costs collected from a clinical trial |
| 2.8 Are the unit costs of resources from the best available source? | Partly | Unit costs appear to be from the trial rather than government sources |
| 2.9 Is an appropriate incremental analysis presented or can it be calculated from the data? | Yes | Incremental costs were calculated |
| 2.10 Are all important parameters whose values are uncertain subjected to appropriate sensitivity analysis? | Yes | PSA was conducted |
| 2.11 Has no potential financial conflict of interest been declared? | Yes | Funding from USAID/MRC |
| 2.12 **Overall assessment:** Minor limitations/potentially serious limitations/very serious limitations  This study was found to have potentially serious limitations due to outcomes data not being reported from the trial. | | |
| Other comments | | |

**Study 18: Reddy et al, 2020.**

| **Section 2: Study limitations** (the level of methodological quality)  This checklist should be used once it has been decided that the study is sufficiently applicable to the context of the guideline | **Yes/partly/no/unclear/NA** | **Comments** |
| --- | --- | --- |
| 2.1 Does the model structure adequately reflect the nature of the topic under evaluation? | Yes | Microsimulation model of HIV disease, treatment, and` transmission, |
| 2.2 Is the time horizon sufficiently long to reflect all important differences in costs and outcomes? | Yes | Lifetime time horizons |
| 2.3 Are all important and relevant outcomes included? | Yes | . All major outcomes, including death, treatment failure, loss to follow-ip, and more are included. |
| 2.4 Are the estimates of baseline outcomes from the best available source? | Yes | from the OFLOTUB RCT |
| 2.5 Are the estimates of relative intervention effects from the best available source? | Yes | from the OFLOTUB RCT |
| 2.6 Are all important and relevant costs included? | Partly | It looks like testing costs have been excluded, but most other relevant costs are present. |
| 2.7 Are the estimates of resource use from the best available source? | Yes | From OFTOFLUB clinical trials |
| 2.8 Are the unit costs of resources from the best available source? | Yes | From South African National Health Laboratory Service |
| 2.9 Is an appropriate incremental analysis presented or can it be calculated from the data? | Yes | ICERs calculated |
| 2.10 Are all important parameters whose values are uncertain subjected to appropriate sensitivity analysis? | Yes | PSA and other analyses were conducted |
| 2.11 Has no potential financial conflict of interest been declared? | Yes | Why? |
| 2.12 **Overall assessment:** Minor limitations/potentially serious limitations/very serious limitations | | |
| Other comments | | |

**Study 19: Bada et al, 2020.**

| **Section 2: Study limitations** (the level of methodological quality)  This checklist should be used once it has been decided that the study is sufficiently applicable to the context of the guideline | **Yes/partly/no/unclear/NA** | **Comments** |
| --- | --- | --- |
| 2.1 Does the model structure adequately reflect the nature of the topic under evaluation? | Yes |  |
| 2.2 Is the time horizon sufficiently long to reflect all important differences in costs and outcomes? | No | Only 9 months (short), so study does not consider long-term costs or outcomes. |
| 2.3 Are all important and relevant outcomes included? | NA | Cost-comparison analysis |
| 2.4 Are the estimates of baseline outcomes from the best available source? | NA | Cost-comparison analysis |
| 2.5 Are the estimates of relative intervention effects from the best available source? | NA | Cost-comparison analysis |
| 2.6 Are all important and relevant costs included? | Yes | All relevant costs across the time horizon are used. |
| 2.7 Are the estimates of resource use from the best available source? | Yes | Yes — based on surveys from costing facilitations. |
| 2.8 Are the unit costs of resources from the best available source? | Partly | Some unit costs from past studies as opposed to government programs |
| 2.9 Is an appropriate incremental analysis presented or can it be calculated from the data? | Yes | Incremental analyses can be calculated. |
| 2.10 Are all important parameters whose values are uncertain subjected to appropriate sensitivity analysis? | NA | Cost-comparison analysis |
| 2.11 Has no potential financial conflict of interest been declared? | Yes | Only funding from research institutes, with funder having no rule in the study. |
| 2.12 **Overall assessment:** Minor limitations/potentially serious limitations/very serious limitations | | |
| Other comments | | |

**Study 20: Diel et al 2015**

| **Section 2: Study limitations** (the level of methodological quality)  This checklist should be used once it has been decided that the study is sufficiently applicable to the context of the guideline | **Yes/partly/no/unclear/NA** | **Comments** |
| --- | --- | --- |
| 2.1 Does the model structure adequately reflect the nature of the topic under evaluation? | Yes | 4 stage Markov model |
| 2.2 Is the time horizon sufficiently long to reflect all important differences in costs and outcomes? | Partly | 10-year time horizons |
| 2.3 Are all important and relevant outcomes included? | Yes | All major outcomes, including death, treatment failure, loss to follow-ip, and more are included. |
| 2.4 Are the estimates of baseline outcomes from the best available source? | Yes | Yes, from RCT |
| 2.5 Are the estimates of relative intervention effects from the best available source? | Yes | Yes, from RCT |
| 2.6 Are all important and relevant costs included? | Yes | Inpatient and outpatient costs are covered |
| 2.7 Are the estimates of resource use from the best available source? | Partly | Came from past analysis which was a Monte Carlo simulation, not an observed RCT |
| 2.8 Are the unit costs of resources from the best available source? | Parly | Came from past analysis which was a Monte Carlo simulation, not an observed RCT |
| 2.9 Is an appropriate incremental analysis presented or can it be calculated from the data? | Yes | ICERs calculated |
| 2.10 Are all important parameters whose values are uncertain subjected to appropriate sensitivity analysis? | Yes | PSA and other analyses were conducted |
| 2.11 Has no potential financial conflict of interest been declared? | Unclear | ? |
| 2.12 **Overall assessment:** Minor limitations/potentially serious limitations/very serious limitations  The study has potentially serious limitations due to poor sourcing of resource use and unit costs, and an improper time horizon. | | |
| Other comments | | |
