# Supplementary material for: A systematic review of economic evaluations of pharmacological treatments for active tuberculosis
Source: Front Public Health. 2024 Apr 16;12:1201512. doi: 10.3389/fpubh.2024.1201512 (PMC11060080; doi:10.3389/fpubh.2024.1201512)
Supplement: Supplementary file 2 [file Data_Sheet_2.doc]

**Search strategies**

| **Database: MEDLINE** |
| --- |
| Database: Ovid MEDLINE(R) <1946 to June 13, 2022>  Search Strategy:  --------------------------------------------------------------------------------  1 exp Tuberculosis/ (202186)  2 (tb* or tubercul* or koch*).tw. (337208)  3 Mycobacterium tuberculosis/ (55472)  4 or/1-3 (377385)  5 exp Antitubercular Agents/ (96328)  6 ((Aminosalicylic adj4 Acid*) or pamisyl* or rezipa*).tw. (3157)  7 (Diarylquinoline* or "Diaryl quinoline*" or Bedaquiline* or Sirturo*).tw. (811)  8 (Ethambutol* or dexambutol* or "emb fatol" or "emb hefa" or emb-fatol or emb-hefa or etibi* or miambutol* or myambutol*).tw. (5141)  9 (ethionamide* or amidazine* or ethioniamide* or trecator*).tw. (993)  10 (isoniazid* or vanillylidenehydrazide* or ftivazide* or hydrazide* or "isonicotinic acid*" or isonex* or phthivazid* or phthivazide* or tubazide*).tw. (21953)  11 (prothionamide* or ektebin* or peteha* or protionamide*).tw. (241)  12 (pyrazinamide* or tisamid*).tw. (3789)  13 (thioacetazon* or ambathizon* or amithiozone* or conteben* or parazone* or thiacetazone*).tw. (589)  14 (Capreomycin* or capastat* or capromycin*).tw. (616)  15 (Cycloserine* or seromycin*).tw. (2573)  16 (Enviomycin* or tuberactinomycin*).tw. (102)  17 ((Mycophenolic adj4 Acid*) or cellcept* or mycophenolate* or myfortic*).tw. (12822)  18 (Rifabutin* or alfacid* or ansamycin* or ansatipin* or mycobutin*).tw. (1806)  19 (rifampin* or benemycin* or rifadin* or rifampicin* or rimactan* or tubocin*).tw. (24202)  20 (viomycin* or celiomycin* or florimycin* or tuberactinomycin* or vinactane* or viocin* or viomicin*).tw. (539)  21 Moxifloxacin/ (2803)  22 (moxifloxacin* or actira* or avalox* or avelox* or izilox* or octegra* or proflox*).tw. (4718)  23 Linezolid/ (3357)  24 (Linezolid* or zyvox*).tw. (5814)  25 (delaminid* or deltyba*).tw. (17)  26 or/5-25 (128153)  27 4 and 26 (56110)  28 animals/ not humans/ (4983617)  29 27 not 28 (53208)  30 limit 29 to english language (36331)  31 limit 30 to ed=20200101-20220614 (4818)  32 Economics/ (27452)  33 exp "Costs and Cost Analysis"/ (258456)  34 Economics, Dental/ (1920)  35 exp Economics, Hospital/ (25586)  36 exp Economics, Medical/ (14341)  37 Economics, Nursing/ (4013)  38 Economics, Pharmaceutical/ (3065)  39 Budgets/ (11615)  40 exp Models, Economic/ (16118)  41 Markov Chains/ (15716)  42 Monte Carlo Method/ (31318)  43 Decision Trees/ (11964)  44 econom$.tw. (292444)  45 cba.tw. (10321)  46 cea.tw. (22773)  47 cua.tw. (1094)  48 markov$.tw. (21483)  49 (monte adj carlo).tw. (34367)  50 (decision adj3 (tree$ or analys$)).tw. (18376)  51 (cost or costs or costing$ or costly or costed).tw. (546565)  52 (price$ or pricing$).tw. (39472)  53 budget$.tw. (27014)  54 expenditure$.tw. (57015)  55 (value adj3 (money or monetary)).tw. (2538)  56 (pharmacoeconomic$ or (pharmaco adj economic$)).tw. (3797)  57 or/32-56 (1081222)  58 Cost-Benefit Analysis/ (89893)  59 (cost* and ((qualit* adj2 adjust* adj2 life*) or qaly*)).tw. (13794)  60 ((incremental* adj2 cost*) or ICER).tw. (14201)  61 (cost adj2 utilit*).tw. (5389)  62 (cost* and ((net adj benefit*) or (net adj monetary adj benefit*) or (net adj health adj benefit*))).tw. (1782)  63 ((cost adj2 (effect* or utilit*)) and (quality adj of adj life)).tw. (18732)  64 (cost and (effect* or utilit*)).ti. (31132)  65 or/58-64 (101952)  66 57 or 65 (1081529)  67 31 and 66 (337) |
| **Notes:** |
|  |

| **Database: EMBASE** |
| --- |
| Database: Embase <1974 to 2022 June 13>  Search Strategy:  --------------------------------------------------------------------------------  1 exp Tuberculosis/ (209853)  2 (tb* or tubercul* or koch*).tw. (419356)  3 Mycobacterium tuberculosis/ (69554)  4 or/1-3 (471693)  5 exp tuberculostatic agent/ (159137)  6 ((Aminosalicylic adj4 Acid*) or pamisyl* or rezipa*).tw. (3518)  7 (Diarylquinoline* or "Diaryl quinoline*" or Bedaquiline* or Sirturo*).tw. (1273)  8 (Ethambutol* or dexambutol* or "emb fatol" or "emb hefa" or emb-fatol or emb-hefa or etibi* or miambutol* or myambutol*).tw. (8265)  9 (ethionamide* or amidazine* or ethioniamide* or trecator*).tw. (1044)  10 (isoniazid* or vanillylidenehydrazide* or ftivazide* or hydrazide* or "isonicotinic acid*" or isonex* or phthivazid* or phthivazide* or tubazide*).tw. (25449)  11 (prothionamide* or ektebin* or peteha* or protionamide*).tw. (402)  12 (pyrazinamide* or tisamid*).tw. (5738)  13 (thioacetazon* or ambathizon* or amithiozone* or conteben* or parazone* or thiacetazone*).tw. (399)  14 (Capreomycin* or capastat* or capromycin*).tw. (725)  15 (Cycloserine* or seromycin*).tw. (2484)  16 (Enviomycin* or tuberactinomycin*).tw. (125)  17 ((Mycophenolic adj4 Acid*) or cellcept* or mycophenolate* or myfortic*).tw. (32752)  18 (Rifabutin* or alfacid* or ansamycin* or ansatipin* or mycobutin*).tw. (2783)  19 (rifampin* or benemycin* or rifadin* or rifampicin* or rimactan* or tubocin*).tw. (35238)  20 (viomycin* or celiomycin* or florimycin* or tuberactinomycin* or vinactane* or viocin* or viomicin*).tw. (272)  21 moxifloxacin/ (22113)  22 (moxifloxacin* or actira* or avalox* or avelox* or izilox* or octegra* or proflox*).tw. (8155)  23 linezolid/ (24689)  24 (Linezolid* or zyvox*).tw. (10646)  25 (delaminid* or deltyba*).tw. (62)  26 or/5-25 (237131)  27 4 and 26 (78967)  28 Nonhuman/ not human/ (5002953)  29 27 not 28 (71160)  30 limit 29 to english language (58794)  31 limit 30 to dc=20200101-20220614 (9096)  32 exp Health Economics/ (960247)  33 exp "Health Care Cost"/ (319557)  34 exp Pharmacoeconomics/ (219019)  35 Monte Carlo Method/ (46365)  36 Decision Tree/ (17538)  37 econom$.tw. (442570)  38 cba.tw. (13595)  39 cea.tw. (38653)  40 cua.tw. (1708)  41 markov$.tw. (35864)  42 (monte adj carlo).tw. (55805)  43 (decision adj3 (tree$ or analys$)).tw. (31191)  44 (cost or costs or costing$ or costly or costed).tw. (901423)  45 (price$ or pricing$).tw. (66351)  46 budget$.tw. (43682)  47 expenditure$.tw. (84262)  48 (value adj3 (money or monetary)).tw. (3935)  49 (pharmacoeconomic$ or (pharmaco adj economic$)).tw. (9260)  50 or/32-49 (2050401)  51 Cost-Benefit Analysis/ (90611)  52 (cost* and ((qualit* adj2 adjust* adj2 life*) or qaly*)).tw. (26776)  53 ((incremental* adj2 cost*) or ICER).tw. (27454)  54 (cost adj2 utilit*).tw. (9860)  55 (cost* and ((net adj benefit*) or (net adj monetary adj benefit*) or (net adj health adj benefit*))).tw. (2796)  56 ((cost adj2 (effect* or utilit*)) and (quality adj of adj life)).tw. (32712)  57 (cost and (effect* or utilit*)).ti. (52436)  58 or/51-57 (156940)  59 50 or 58 (2050851)  60 31 and 59 (616) |
| **Notes:** |
|  |

| **Database: CDSR** |
| --- |
| #1 MeSH descriptor: [Tuberculosis] explode all trees 2503  #2 (tb* or tubercul* or koch*) 43202  #3 MeSH descriptor: [Mycobacterium tuberculosis] this term only 358  #4 {OR #1-#3} 43203  #5 MeSH descriptor: [Antitubercular Agents] explode all trees 2022  #6 ((Aminosalicylic near/4 Acid*) or pamisyl* or rezipa*) 659  #7 (Diarylquinoline* or "Diaryl quinoline*" or Bedaquiline* or Sirturo*) 104  #8 (Ethambutol* or dexambutol* or "emb fatol" or "emb hefa" or emb-fatol or emb-hefa or etibi* or miambutol* or myambutol*) 783  #9 (ethionamide* or amidazine* or ethioniamide* or trecator*) 83  #10 (isoniazid* or vanillylidenehydrazide* or ftivazide* or hydrazide* or "isonicotinic acid*" or isonex* or phthivazid* or phthivazide* or tubazide*) 1894  #11 (prothionamide* or ektebin* or peteha* or protionamide*) 53  #12 (pyrazinamide* or tisamid*) 815  #13 (thioacetazon* or ambathizon* or amithiozone* or conteben* or parazone* or thiacetazone*) 78  #14 (Capreomycin* or capastat* or capromycin*) 40  #15 (Cycloserine* or seromycin*) 491  #16 (Enviomycin* or tuberactinomycin*) 3  #17 ((Mycophenolic near/4 Acid*) or cellcept* or mycophenolate* or myfortic*) 4712  #18 (Rifabutin* or alfacid* or ansamycin* or ansatipin* or mycobutin*) 283  #19 (rifampin* or benemycin* or rifadin* or rifampicin* or rimactan* or tubocin*) 2759  #20 (viomycin* or celiomycin* or florimycin* or tuberactinomycin* or vinactane* or viocin* or viomicin*) 12  #21 MeSH descriptor: [Moxifloxacin] this term only 910  #22 (moxifloxacin* or actira* or avalox* or avelox* or izilox* or octegra* or proflox*) 1812  #23 MeSH descriptor: [Linezolid] this term only 246  #24 (Linezolid* or zyvox*) 581  #25 (delaminid* or deltyba*) 3  #26 {OR #5-#25} 12957  #27 #4 and #26 with Cochrane Library publication date Between Jan 2020 and Jun 2022, in Cochrane Reviews, Cochrane Protocols 24 |

| **Database: EconLit** |
| --- |
| Database: Econlit <1886 to June 09, 2022>  Search Strategy:  --------------------------------------------------------------------------------  1 (tb* or tubercul* or koch*).tw. (1026)  2 ((Aminosalicylic adj4 Acid*) or pamisyl* or rezipa*).tw. (0)  3 (Diarylquinoline* or "Diaryl quinoline*" or Bedaquiline* or Sirturo*).tw. (1)  4 (Ethambutol* or dexambutol* or "emb fatol" or "emb hefa" or emb-fatol or emb-hefa or etibi* or miambutol* or myambutol*).tw. (0)  5 (ethionamide* or amidazine* or ethioniamide* or trecator*).tw. (0)  6 (isoniazid* or vanillylidenehydrazide* or ftivazide* or hydrazide* or "isonicotinic acid*" or isonex* or phthivazid* or phthivazide* or tubazide*).tw. (2)  7 (prothionamide* or ektebin* or peteha* or protionamide*).tw. (0)  8 (pyrazinamide* or tisamid*).tw. (0)  9 (thioacetazon* or ambathizon* or amithiozone* or conteben* or parazone* or thiacetazone*).tw. (0)  10 (Capreomycin* or capastat* or capromycin*).tw. (1)  11 (Cycloserine* or seromycin*).tw. (0)  12 (Enviomycin* or tuberactinomycin*).tw. (0)  13 ((Mycophenolic adj4 Acid*) or cellcept* or mycophenolate* or myfortic*).tw. (2)  14 (Rifabutin* or alfacid* or ansamycin* or ansatipin* or mycobutin*).tw. (0)  15 (rifampin* or benemycin* or rifadin* or rifampicin* or rimactan* or tubocin*).tw. (1)  16 (viomycin* or celiomycin* or florimycin* or tuberactinomycin* or vinactane* or viocin* or viomicin*).tw. (0)  17 (moxifloxacin* or actira* or avalox* or avelox* or izilox* or octegra* or proflox*).tw. (1)  18 (Linezolid* or zyvox*).tw. (1)  19 (delaminid* or deltyba*).tw. (0)  20 or/2-19 (7)  21 1 and 20 (1)  22 limit 21 to yr="2020 -Current" (0) |
| **Notes:** |
|  |

| **Database: INAHTA** |
| --- |
| ((tb* or tubercul* or koch*))[Title] OR ((tb* or tubercul* or koch*))[abs] |
